# Global, regional, and national burden of heart failure and its underlying causes, 1990–2021: results from the global burden of disease study 2021

**DOI:** 10.1186/s40364-025-00728-8

**Published:** 2025-01-23

**Authors:** Jun Ran, Ping Zhou, Jinxi Wang, Xuemei Zhao, Yan Huang, Qiong Zhou, Mei Zhai, Yuhui Zhang

**Affiliations:** 1 State Key Laboratory of Cardiovascular Disease, Heart Failure Center, National Center for Cardiovascular Diseases, Fuwai Hospital, Chinese Academy of Medical Sciences and Peking Union Medical College, Beijing, China; 2 Fuwai Hospital, National Center for Cardiovascular Diseases, No. 167 Beilishi Road, Beijing, 10037 China

**Keywords:** Heart failure, Global burden of disease, Epidemiology, Socio-demographic Index, Prevalence, Years lived with disability

## Abstract

**Background:**

Heart failure (HF) remains a significant public health challenge globally. This study aims to systematically analyze the global HF disease burden from 1990 to 2021 across temporal, spatial, and demographic dimensions to provide evidence for targeted prevention and control strategies.

**Methods:**

Using data from the Global Burden of Disease (GBD) 2021 study, we analyzed the global HF burden through prevalent cases, years lived with disability (YLDs), and age-standardized rates per 100,000 population. Temporal trends were evaluated using estimated annual percentage change (EAPC) and joinpoint regression analysis. The relationship between the Socio-demographic Index (SDI) and disease burden was explored through Pearson correlation analysis, while attribution analysis identified the main causes of HF. When appropriate, analyses were stratified by 5 SDI regions, 21 GBD regions, 204 countries and territories, 20 age groups, and both sexes.

**Results:**

Global HF prevalence and YLDs burden showed substantial increases from 1990 to 2021, with age-standardized prevalence increasing from 641.14 to 676.68 per 100,000 population. Notably, high-SDI regions exhibited a declining burden since 2019, indicating a potential global turning point. High-income North America bears the heaviest burden while South Asia shows the fastest growth rate. The correlation between disease burden and SDI level was negligible. The disease burden in males consistently exceeded that in females, with prevalence and YLDs rates rising sharply after age 60. The main causes and their attributable proportions were: ischemic heart disease (34.53%), hypertensive heart disease (22.53%), other cardiomyopathies (7.61%), chronic obstructive pulmonary disease (6.51%), and congenital heart anomalies (5.69%), with their distribution patterns differing across age groups and regions.

**Conclusion:**

Global burden of HF increased significantly over recent decades, with a potential turning point in 2019 and marked regional disparities. It is essential to prioritize regions with heavy burdens or rapid growth rates, strengthen the management of major causes, and monitor HF burden trends in the post-COVID era.

**Supplementary Information:**

The online version contains supplementary material available at 10.1186/s40364-025-00728-8.

## Introduction

Heart failure (HF), a severe manifestation or end-stage of various cardiac diseases, has become a major public health concern due to its high mortality and rehospitalization rates. Recent years have witnessed significant, even breakthrough, advancements in the diagnosis and treatment of this critical condition. Several international heart failure associations jointly issued a universal definition and classification standard for HF [1], standardizing the refined diagnosis and classification of HF and laying the foundation for precision treatment. Novel pharmacological agents such as ARNI (Angiotensin Receptor-Neprilysin Inhibitor), SGLT2 (Sodium-Glucose Cotransporter-2) inhibitors, and sGC (soluble Guanylate Cyclase) stimulators have shown promising results in HF treatment, with their applications continually expanding [2–5]. Mechanical assist devices like left ventricular assist devices (LVADs) are rapidly evolving [6–9], while management strategies for acute HF and HF comorbidities continue to be optimized. In response to these advancements, multiple global heart failure associations have successively updated and released heart failure diagnosis and treatment guidelines to provide the latest guidance for clinical practice [10–12].

However, as a significant global public health concern, trends in the global burden of HF remain controversial: some studies have indicated an upward trend in HF burden over recent decades [13–15], while others have reported declining patterns [16, 17]. Moreover, substantial regional disparities exist in disease burden, and some regions still lack reliable epidemiological data. These factors pose significant challenges for comprehensive assessment and the development of targeted prevention and control strategies.

Given the rapid developments in HF diagnosis and treatment, as well as these inconsistent epidemiological findings, a systematic analysis of the most recent global disease burden of HF is warranted. This analysis is crucial to evaluate the actual effectiveness of diagnostic and therapeutic advances and to provide a scientific basis for subsequent formulation of targeted prevention and control strategies, as well as optimization of medical resource allocation. In May 2024, the Global Burden of Disease (GBD) database released the latest global disease burden data for 2021, providing an opportune moment to reassess the global burden of HF.

This study will utilize the most recent GBD data to systematically analyze the temporal, spatial, and demographic distribution characteristics of the global HF disease burden (prevalence and years lived with disability [YLDs]) from 1990 to 2021. Additionally, it will conduct an attribution analysis of the main causes of HF. The aim is to provide a reference for a deeper understanding of the current global disease burden of HF and to offer a scientific basis for developing targeted prevention and control strategies and optimizing the allocation of medical resources.

## Method

### GBD overview

The GBD study stands as the most comprehensive scientific endeavor to assess global health trends. Spearheaded by the University of Washington’s Institute for Health Metrics and Evaluation (IHME), this initiative engages over 12,000 researchers from more than 160 nations and territories.

GBD 2021 [18–20] utilized 100,983 global data sources, including 19,189 new additions. These encompassed scientific literature, surveys, surveillance data, registries, and clinical informatics for non-fatal data, while fatal data sources comprised vital registration, verbal autopsies, surveys, censuses, and specialized records.

GBD 2021 provides estimates for 371 diseases and injuries across 204 countries and territories, spanning 25 age groups and both sexes from 1990 to 2021. The study reports on various health metrics, including prevalence, incidence, mortality, years lived with disability (YLDs), years of life lost (YLLs), disability-adjusted life-years (DALYs), and healthy life expectancy (HALE).

Metrics were calculated as counts, rates per 100,000 population (both all-age and age-specific), and age-standardized rates using the GBD standard population structure. Uncertainty was propagated throughout the estimation process, with each metric computed through 500 simulation draws. Final estimates represent the mean across these simulations, with 95% uncertainty intervals (UIs) derived from the 2.5th and 97.5th percentiles.

Additionally, GBD 2021 examines burden across Socio-demographic Index (SDI) quintiles. The SDI is a composite measure incorporating income per capita, average years of schooling, and fertility rates among females under 25 for each location. SDI scores range from 0 to 100, with higher scores indicating better socioeconomic conditions.

The comprehensive data from GBD 2021 is publicly available and can be downloaded at [https://vizhub.healthdata.org/gbd-results/] [21].

### Heart failure and attributable causes

#### Heart failure definition

The GBD case definition for HF impairment data sources includes studies in which HF was diagnosed clinically using structured criteria such as the Framingham or European Society of Cardiology criteria. Since 2016, the study used Stage C and above as defined in the Universal Definition of HF to capture both persons who are currently symptomatic and those who have been diagnosed with HF but are currently asymptomatic.

#### Attributable causes definition

HF can be caused by various diseases. In the GBD 2021 study, 27 most-detailed causes were included in the causal attribution analysis for HF. These 27 causes include: ischemic heart disease, hypertensive heart disease, chronic obstructive pulmonary disease (COPD), interstitial lung disease and pulmonary sarcoidosis, pulmonary arterial hypertension, various pneumoconioses, rheumatic heart disease, congenital heart anomalies, various valvular diseases, myocarditis, various cardiomyopathies, substance use disorders, Chagas disease, endocrine disorders, hematological disorders, other cardiovascular and circulatory diseases, atrial fibrillation, chronic kidney disease, cirrhosis and other chronic liver diseases, and acute and chronic stroke.

The selection criteria for these causes encompassed: (1) Literature review to identify diseases proven to cause clinical HF; (2) Quantitative analysis of death certificates and hospital records from 93 country-years to identify diseases most commonly co-occurring with HF; (3) Expert opinion on diseases that lead to clinical HF; (4) These causes represent common, clinically relevant, and quantifiable entities that can be stably estimated; (5) Inclusion of an “other” category to ensure estimates are collectively exhaustive and mutually exclusive.

Detailed definitions for each cause can be found in the relevant GBD study articles [19, 20].

#### GBD modelling strategy and burden estimation

The GBD study employs a sophisticated methodology to estimate HF prevalence, etiological distribution, and YLDs. Overall HF prevalence is estimated using DisMod-MR 2.1 (Disease Modeling-Meta Regression tool version 2.1), which integrates literature, hospital, and claims data, with the Healthcare Access and Quality Index serving as a key covariate. Etiology-specific estimations involve separate modeling for certain causes and utilize the relationship between prevalence, cause-specific mortality rate, and excess mortality rate, modeled using MR-BRT (Meta-regression—Bayesian, Regularized, Trimmed). The study estimates and scales seven broad cause groupings, followed by additional scaling for specific etiologies. Severity distribution, crucial for YLDs calculation, is primarily based on Medical Expenditure Panel Survey data. YLDs are estimated by applying disability weights to severity-specific prevalence estimates (asymptomatic, mild, moderate, and severe), followed by comorbidity adjustment. This comprehensive approach integrates diverse data sources and advanced statistical techniques to provide a thorough assessment of HF’s prevalence and disability burden, thereby informing global health policies and research priorities.

The detailed modelling strategy can be found in the official GBD methodological appendix at [https://www.healthdata.org/gbd/methods-appendices-2021] [22].

### Statistical analysis for this study

#### General principles and statistical software

This study employed R statistical software (version 4.4.0) for data analysis and visualization. while the joinpoint regression analyses were conducted using Joinpoint Regression Program version 5.3.0 [23], developed by the National Cancer Institute (United States).The statistical significance level was set at *p* < 0.05 for all analyses.

#### Estimated annual percentage change (EAPC)

To quantify the overall long-term trends, we calculated the EAPC by fitting a linear regression to the natural logarithm of annual rates. Years were coded sequentially as t = 0, 1, 2, …, 31 (with 1990 as the index year). The regression model was specified as: ln(Rt) = α + βt + ε

$$\:\text{ln}\left(\text{Rt}\right)\:={\alpha}+\beta\;\text{t}+\varepsilon$$

where Rt represents the rate in year t, α denotes the intercept, β is the regression coefficient, and ε is the error term. The EAPC was calculated as: EAPC = (e^β - 1) × 100%

$$\:EAPC\:=\:(e^\wedge{\beta}-1)\times100\%$$

with 95% confidence intervals derived from the standard error of β. The trend was considered statistically significant if the confidence interval excluded zero.

#### Joinpoint regression analysis

To identify significant changes in temporal trends and characterize recent pattern changes, we conducted joinpoint regression analysis using the Joinpoint Regression Program (version 5.3.0, National Cancer Institute, United States). This method identifies points (joinpoints) where the linear trend changes significantly in magnitude or direction, providing a more nuanced understanding of temporal patterns compared to the single summary measure of EAPC.

The joinpoint analysis proceeded as follows: (1) The program tested the optimal number of joinpoints (up to a maximum of 5) needed to characterize the trend, starting with the null hypothesis of zero joinpoints. (2) For each time segment between joinpoints, the Annual Percent Change (APC) was calculated based on the linear regression of logarithmically transformed data using the formula: APC = (e^β- 1)×100%, where β represents the slope coefficient from the segmented regression. (3) The selection of the final model was based on the Monte Carlo Permutation method, testing the significance of joinpoints with a threshold of *p* < 0.05. (4) The analysis yielded: The optimal number and temporal location of joinpoints, Segment-specific APCs with corresponding 95% confidence intervals, Tests for parallelism between different series where applicable.

#### Pearson correlation analysis

Pearson correlation coefficients (r) were calculated to assess the relationships between SDI and incidence rate, death rate, and Disability-Adjusted Life Years (DALY) rate. The formula for Pearson’s r is: r = Σ[(xi - x̄)(yi - ȳ)] / √[Σ(xi - x̄)^2 Σ(yi - ȳ)^2], where xi and yi are the observed values of the two variables, and x̄ and ȳ are their respective means. The r value ranges from − 1 to 1, with absolute values closer to 1 indicating stronger correlations. Positive values denote positive correlations, while negative values indicate inverse relationships. P-values < 0.05 were considered statistically significant.

## Results

### Prevalence burden

**Fig. 1:**
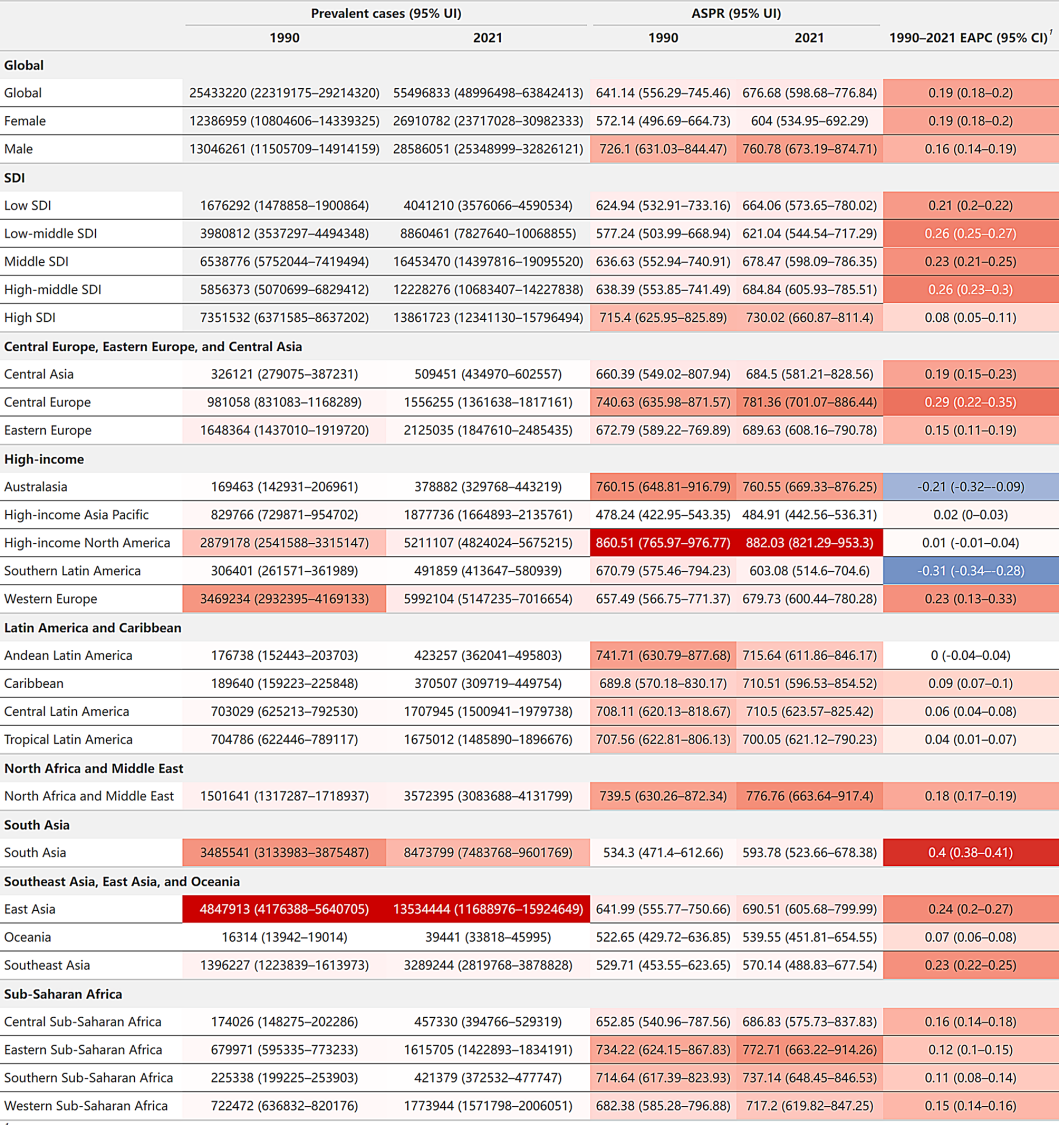
The prevalence burden of HF and its temporal trends by regions, 1990–2021 Note: ¹Color intensity represents value magnitude. Red indicates positive values, darker for larger. Blue indicates negative values, darker for more negative. Abbreviations: HF = heart failure; ASPR = age-standardized prevalence (per 100,000 population); HF = heart failure; UI = uncertainty interval; EAPC = estimated annual percentage change; CI = confidence interval; SDI = socio-demographic index

**Fig. 2:**
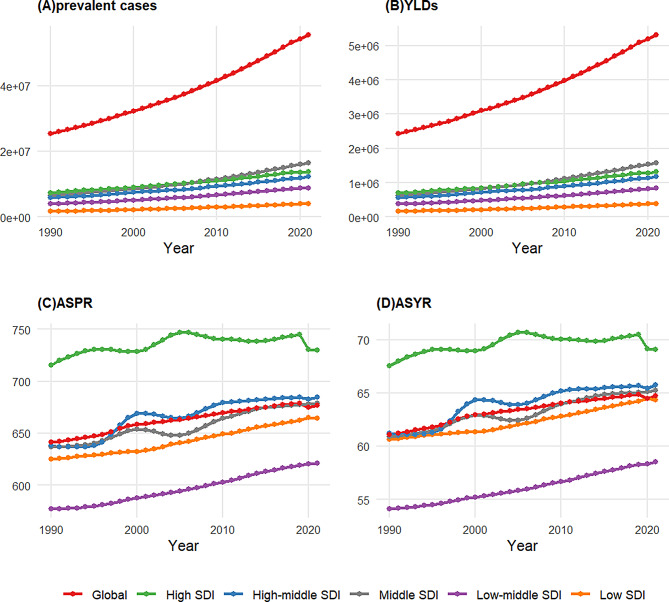
Temporal trends of HF burdens across different SDI regions from 1990 to 2021 **(A)** Prevalent cases of HF across different SDI regions from 1990 to 2021, for all ages and both sexes. **(B)** YLDs of HF across different SDI regions from 1990 to 2021, for all ages and both sexes. **(C)** ASPR of HF across different SDI regions from 1990 to 2021, for both sexes. **(D)** ASYR of HF across different SDI regions from 1990 to 2021, for both sexes. Note: Vertical axes use scientific notation (e.g., 5e + 06 = 5,000,000) for large numbers. Abbreviations: HF = heart failure; SDI = socio-demographic index; YLDs = years lived with disability; ASYR = age-standardized YLDs rate (per 100,000 population); ASPR = age-standardized prevalence (per 100,000 population)

From 1990 to 2021, the global prevalence of HF more than doubled, increasing from 25.43 million cases (95% UI: 22.32–29.21 million) to 55.50 million cases (95% UI: 49.00-63.84 million). Even after adjusting for population age structure, the age-standardized prevalence (ASPR) showed an overall upward trend, from 641.14 (95% UI: 556.29-745.46) to 676.68 (95% UI: 598.68-776.84) per 100,000, with an EAPC of 0.19 (95% CI: 0.18–0.2) (Figs. 1 and 2A/2 C). Notably, within this overall ascending pattern, joinpoint regression analysis identified a recent shift in trends: High SDI regions demonstrated a significant decline from 2019 to 2021 (APC: -1.07%, *p* < 0.05), while other SDI regions, although still increasing, showed a decelerated growth rate. These changes culminated in an overall declining trend in global ASPR from 2018 to 2021 (APC: -0.13%, *p* < 0.05), marking a potential turning point in the global prevalence burden of HF (Figure S1).

In 2021, high-income North America had the highest ASPR, reaching 882.03 per 100,000 (95% UI: 821.29–953.3). However, temporal trends showed that some high-income regions, such as North America, Australasia, and Southern Latin America, showed very low or negative EAPCs despite their high ASPRs, indicating that HF prevalence in these regions has stabilized or begun to decline. In contrast, South Asia, although having a relatively low ASPR (593.78 per 100,000, 95% UI: 523.66-678.38), showed the highest EAPC (0.4, 95% CI: 0.38–0.41), indicating the fastest growth in disease burden and requiring special attention. East Asia, with its large population base, showed an extremely high total prevalence (13.5 million, 95% UI: 11.7–15.9) despite a moderate ASPR (690.51 per 100,000, 95% UI: 605.68-799.99). Its relatively high EAPC (0.24, 95% CI: 0.2–0.27) indicates continuing challenges (Fig. 1).

**Fig. 3:**
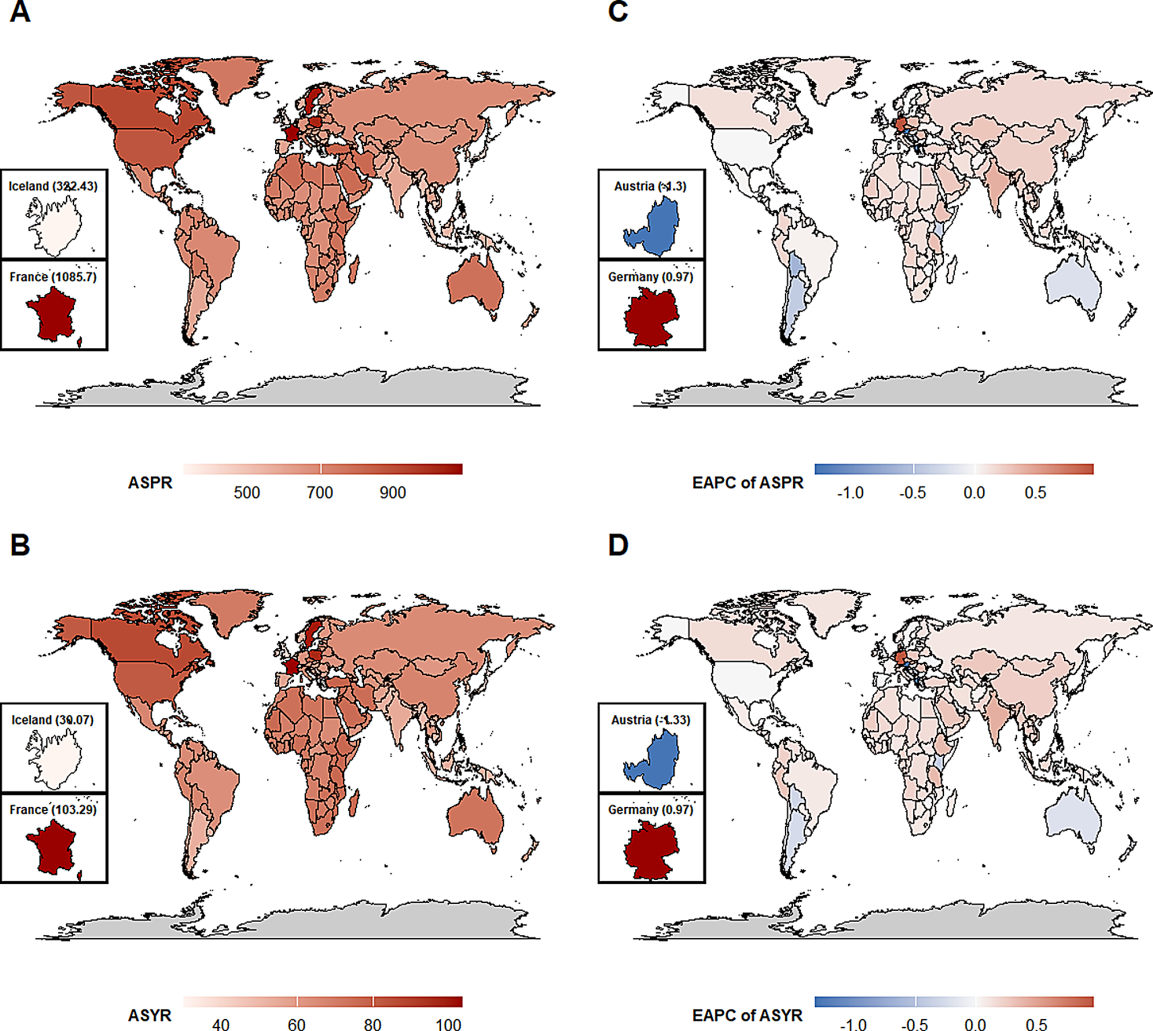
Geographic distributions of HF burdens and their EAPCs across 204 countries and territories **(A)** ASPR of HF in 2021, for both sexes. Bottom-left small maps display the highest (France) and lowest (Iceland) rates. **(B)** ASYR of HF in 2021, for both sexes. Bottom-left small maps display the highest (France) and lowest (Iceland) rates. **(C)** EAPC of ASPR between 1990 and 2021, for both sexes. Bottom-left small maps display the most positive (Germany) and negative (Austria) changes. **(D)** EAPC of ASYR between 1990 and 2021, for both sexes. Bottom-left small maps display the most positive (Germany) and negative (Austria) changes. Note: Darker shades of red in panels A-B indicate higher burden. In panels C-D, blue shades represent decreases in disease burden, while red shades represent increases over time. Gray areas indicate regions with insufficient data. Abbreviations: ASPR = age-standardized prevalence (per 100,000 population); HF = heart failure; YLDs = years lived with disability; ASYR = age-standardized YLDs rate (per 100,000 population); EAPC = estimated annual percentage change

Notably, Western Europe, a high-income region, exhibited significant intra-regional differences in prevalence burden. France and Sweden showed the world’s highest ASPR and relatively low EAPC: France had an ASPR of 1085.7 per 100,000 (95% UI: 920.21-1284.19) with an EAPC of only 0.01 (95% CI: -0.11-0.14), while Sweden showed an ASPR of 1060.21 per 100,000 (95% UI: 936.14-1219.74) and an EAPC of -0.01 (95% CI: -0.09-0.07). In contrast, Germany had a relatively low ASPR (675.91 per 100,000, 95% UI: 574.85–799.7) but the world’s highest EAPC (0.97, 95% CI: 0.68–1.25). Conversely, Austria, also in Western Europe, had the lowest EAPC (-1.3, 95% CI: -1.44–1.16) globally. These findings reveal substantial variations in prevalence burden even among regions with similar high-income status (Fig. 3A and C; Figure S3A; Table S1).

When using SDI to measure socioeconomic development, although high-SDI and high-middle SDI regions generally had higher HF prevalence burden than low and low-middle SDI regions (Fig. 2C), correlation analysis revealed a negligible relationship between SDI and prevalence burden (|R| < 0.2)(Figure S4A, Figure S5A).

**Fig. 4:**
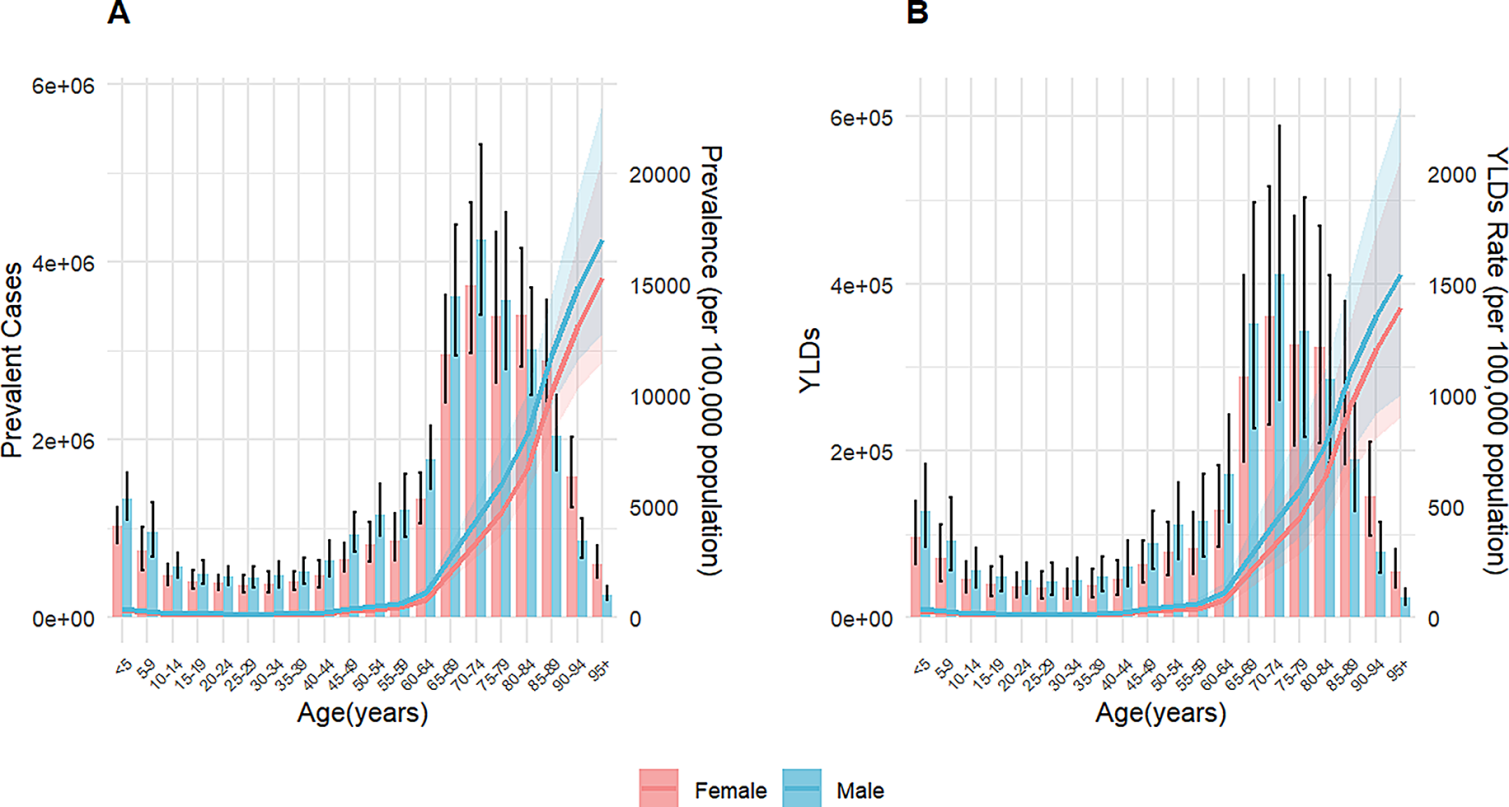
Global trends of HF burdens across age groups by sex, 2021 **(A)** Prevalent cases and prevalence of HF across age groups by sex. **(B)** YLDs and YLDs rate of HF across different age groups by sex. Note: Bars represent absolute numbers (left y-axis), while lines represent rates (right y-axis). Vertical black lines indicate 95% uncertainty intervals. Abbreviations: HF = heart failure; YLDs = years lived with disability

Regarding gender differences, the prevalence burden increased in both sexes, with males showing higher ASPRs than females. In 2021, the ASPR for males was 760.78 per 100,000 (95% UI: 673.19-874.71), compared to 604 per 100,000 (95% UI: 534.95-692.29) for females (Fig. 1; Figure S7A). Age distribution analysis showed that HF prevalence increased with age, with a sharp rise after 60 years old. Although prevalence continued to climb in the 80 + age group, the absolute number of cases decreased due to a smaller population base (Fig. 4A). This age distribution trend was similar across different SDI regions (Figure S6A).

### YLDs burden

The YLDs burden of HF shows similar distribution patterns to its prevalence.

**Fig. 5:**
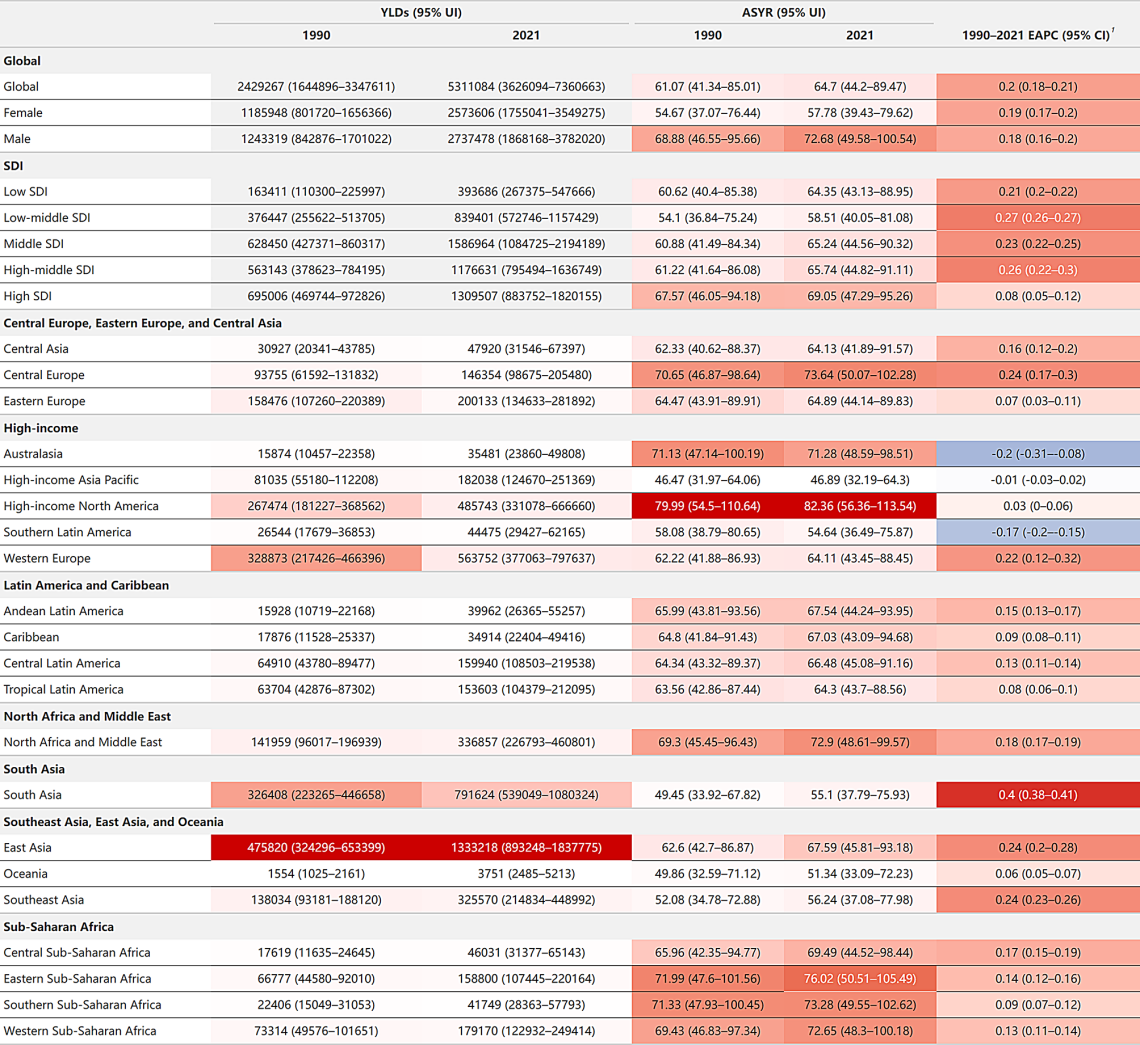
The YLDs burden of HF and its temporal trends by regions, 1990–2021 Note: ¹Color intensity represents value magnitude. Red indicates positive values, darker for larger. Blue indicates negative values, darker for more negative. Abbreviations: HF = heart failure; YLDs = years lived with disability; ASYR = age-standardized YLDs rate (per 100,000 population); UI = uncertainty interval; EAPC = estimated annual percentage change; CI = confidence interval; SDI = socio-demographic index

From 1990 to 2021, the global YLDs due to HF more than doubled, from 2.43 million years (95% UI: 1.64–3.35) to 5.31 million years (95% UI: 3.63–7.36). The age-standardized YLDs rate (ASYR) increased from 61.07 (95% UI: 41.34–85.01) to 64.7 per 100,000 (95% UI: 44.2-89.47), with an EAPC of 0.2 (95% CI: 0.18–0.21) (Figs. 2B and 5/2D). Notably, within this overall ascending pattern, joinpoint regression analysis identified a recent shift in trends: High SDI regions demonstrated a significant decline from 2019 to 2021 (APC: -1.09%, *p* < 0.05), while other SDI regions, although still increasing, showed a decelerated growth rate. These changes culminated in an overall declining trend in global ASYR from 2018 to 2021 (APC: -0.11%, *p* < 0.05), suggesting a potential turning point (Figure S2).

As for regional distribution in 2021, High-income North America had the highest ASYR, reaching 82.36 per 100,000 (95% UI: 56.36-113.54). However, temporal trends showed that some high-income regions, such as high-income North America and Australasia, despite having high ASYRs, showed low or even negative EAPCs, indicating stabilization or decline in their heart failure YLDs burden. In contrast, South Asia, despite having a relatively low ASYR (55.1 per 100,000, 95% UI: 37.79–75.93), had the highest EAPC (0.4, 95% CI: 0.38–0.41), suggesting the fastest growth in YLDs burden and warranting special attention. East Asia, due to its large population base, had an extremely high total YLDs (1.33 million, 95% UI: 0.89–1.84) despite a moderate ASYR (67.59 per 100,000, 95% UI: 45.81–93.18). Its relatively high EAPC (0.24, 95% CI: 0.2–0.28) indicates continuing challenges (Fig. 5).

Notably, Western Europe, a high-income region, exhibited significant intra-regional differences in YLDs burden. France and Sweden exemplified the world’s highest ASYR and relatively low EAPC: France had an ASYR of 103.29 per 100,000 (95% UI: 67.32-143.42), but an EAPC of only 0.04 (95% CI: -0.09-0.16), indicating burden stabilization. Similarly, Sweden showed an ASYR of 99.87 per 100,000 (95% UI: 66.88-137.67) and an EAPC of -0.03 (95% CI: -0.11-0.05), suggesting a slight downward trend. In contrast, Germany had a moderate ASYR (63.39 per 100,000, 95% UI: 40.85–88.58) but the world’s highest EAPC (0.97, 95% CI: 0.68–1.25), indicating a rapidly increasing YLDs burden. Conversely, Austria, also in Western Europe, had the lowest EAPC (-1.33, 95% CI: -1.47–1.18) globally. These findings highlight substantial regional variations in YLDs burden even among regions sharing high-income status (Table S2; Fig. 3B/3D; Figure S3B).

When using SDI to measure socioeconomic development, although temporal trends showed that high-SDI and high-middle SDI regions generally had higher heart failure YLDs burdens than low and low-middle SDI regions (Fig. 2D), correlation analysis revealed a negligible relationship between SDI and YLDs burden(|R| < 0.2). (Figure S4B, Figure S5B)

Regarding gender differences, the YLDs burden increased in both sexes, with males showing slightly higher ASYRs than females (Fig. 5; Figure S7B). Heart failure YLDs rates increased with age, showing a sharp rise after 60 years old. Although YLDs rates continued to climb in the 80 + age group, the absolute number of YLDs decreased due to a smaller population base (Fig. 4B). This age distribution trend was similar across different SDI regions (Figure S6B).

### Attribution analysis

**Fig. 6:**
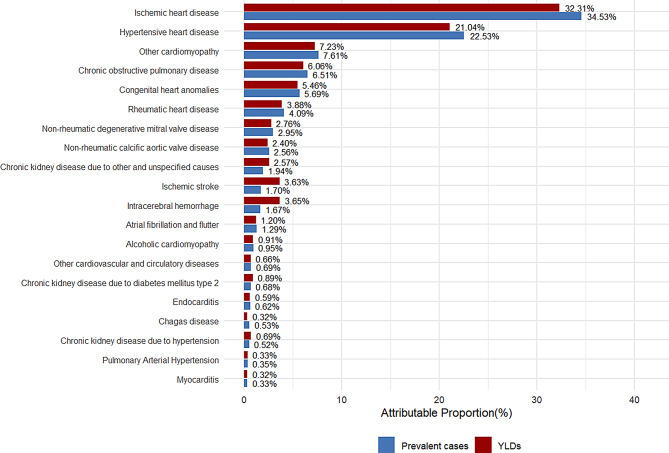
Proportion of HF prevalent cases and YLDs attributable to major underlying causes globally, 2021. Abbreviations: HF = heart failure; YLDs = years lived with disability

In 2021, the main causes of global HF prevalence and their attributable proportions were: ischemic heart disease (34.53%), hypertensive heart disease (22.53%), other cardiomyopathies (7.61%), chronic obstructive pulmonary disease (6.51%), and congenital heart anomalies (5.69%) (Fig. 6). From 1990 to 2021, the composition of major causes remained largely unchanged (Figure S10A). The ranking of top contributing causes was similar for both males and females (Figure S11).

**Fig. 7:**
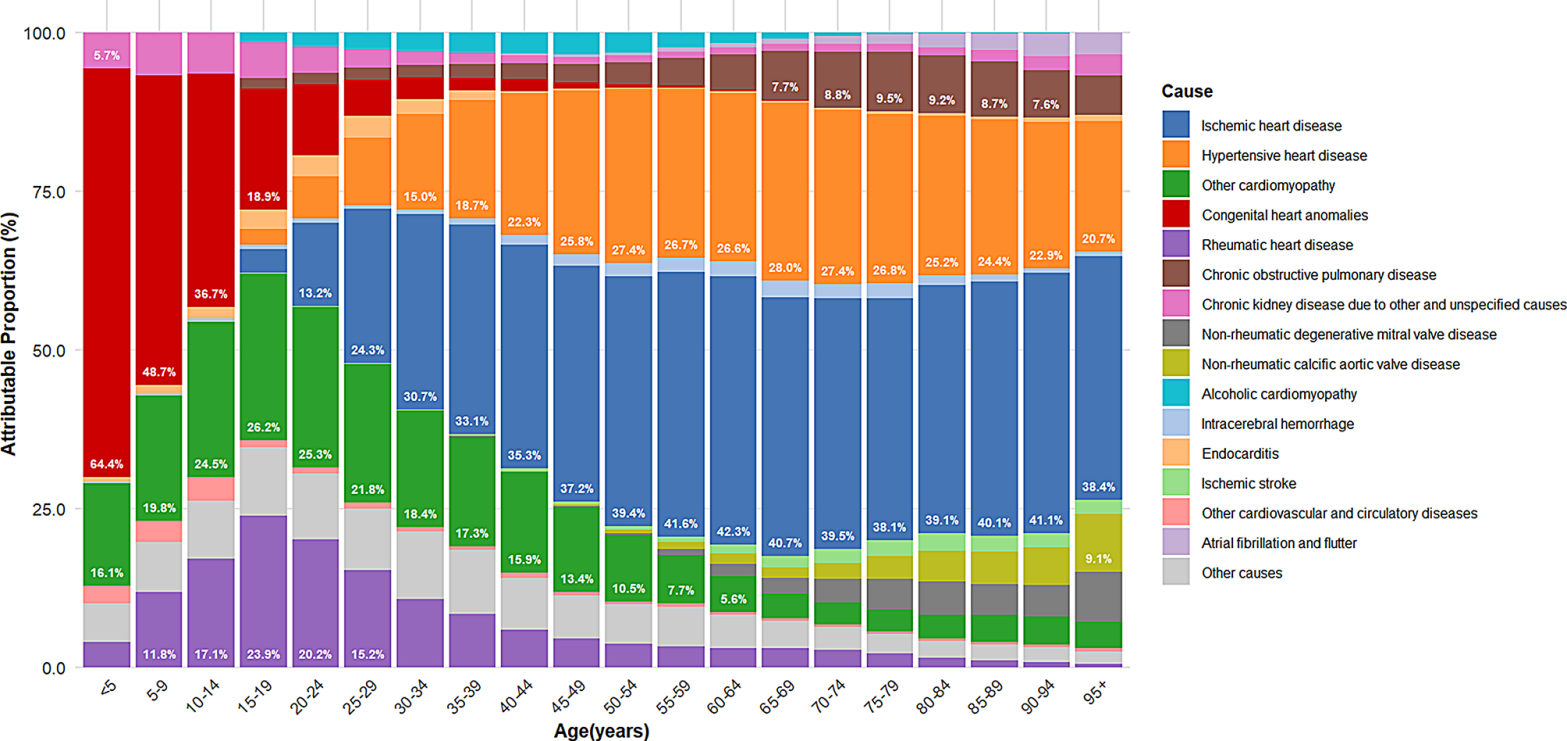
Proportion of HF prevalent cases attributable to major underlying causes by age groups globally, 2021. Abbreviations: HF = heart failure

HF etiology showed a clear age-related pattern (Fig. 7). Among children and adolescents, congenital heart anomalies were the primary cause, accounting for up to 64.4% in the under-5 age group. In adolescents, other cardiomyopathies and rheumatic heart disease were prominent contributors, peaking in the 15–19 age group at 26.2% and 23.9%, respectively. With advancing age, ischemic heart disease and hypertensive heart disease gradually became the main causes of HF prevalence. In the 60 + age group, ischemic heart disease accounted for approximately 40% of attributions, while hypertensive heart disease contributed about 25–28%. In those aged 65 and above, the contributions of chronic obstructive pulmonary disease and non-rheumatic valvular heart disease were noteworthy. This age-related pattern of causal distribution was similar across genders (Figure S8). The age distribution of YLDs attribution closely matched that of prevalence (Figure S9).

**Fig. 8:**
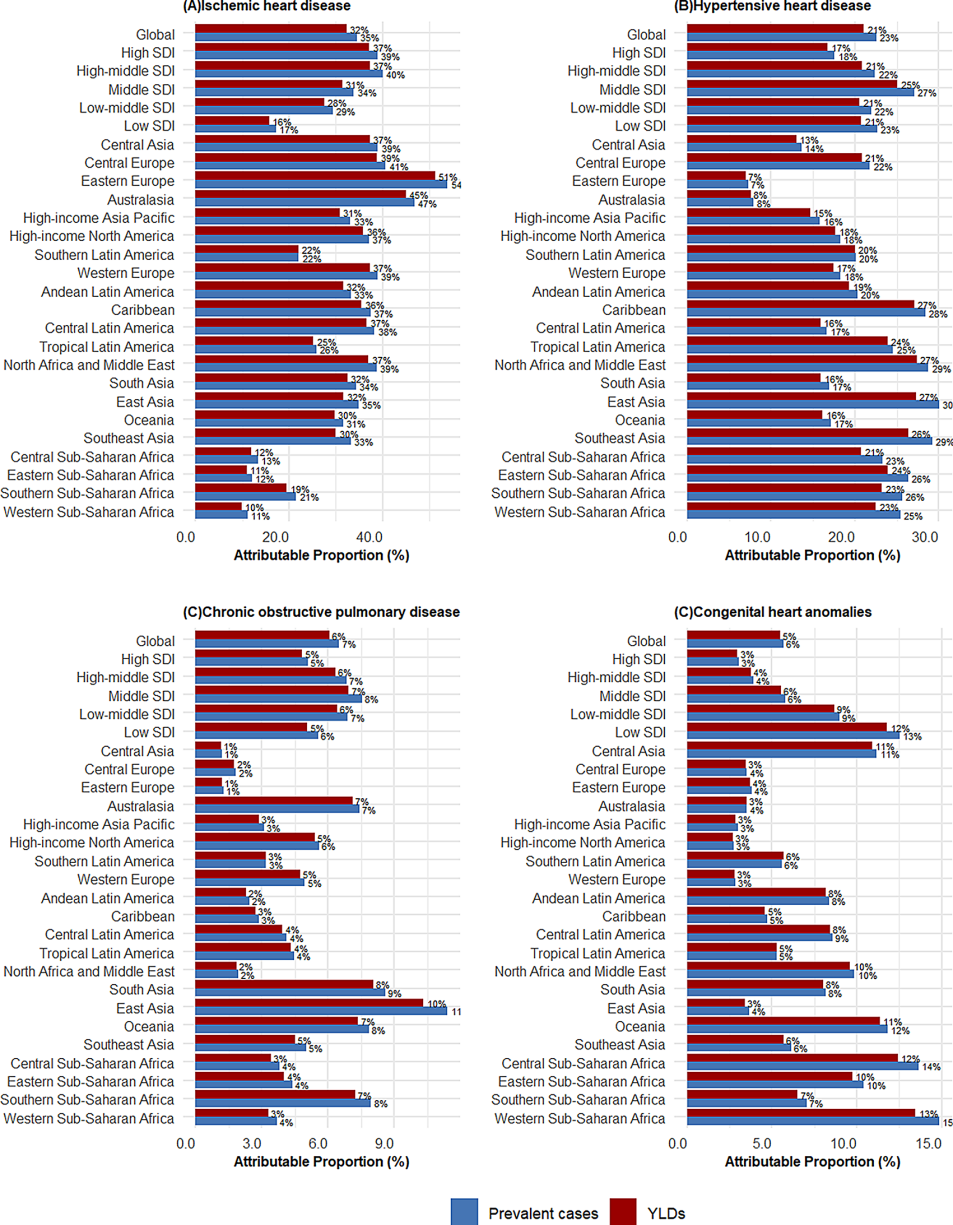
Proportion of HF prevalent cases and YLDs attributable to major causes globally and regionally, 2021. Abbreviations: HF = heart failure; SDI = socio-demographic index; YLDs = years lived with disability

Regional differences existed in causal attribution. Ischemic heart disease accounted for over 20% of HF causes in all regions except sub-Saharan Africa, with Eastern Europe showing the highest attribution at 54.0%. In sub-Saharan Africa, hypertensive heart disease accounted for about a quarter of HF cases, while in East Asia, it accounted for 30.0%. Chronic obstructive pulmonary disease contributed significantly in East Asia, accounting for 11.4% of HF prevalence (Fig. 8). This regional heterogeneity in HF causes provides evidence for targeted prevention and intervention strategies.

## Dicussion

This study analyzed the global HF burden using the latest GBD data. Our main findings are as follows: (1) Global HF prevalence and YLDs burden showed substantial increases from 1990 to 2021, with ASPR increasing from 641.14 to 676.68 per 100,000. Notably, high-SDI regions exhibited a declining burden since 2019, indicating a potential global turning point. (2) High-income North America bears the heaviest burden while South Asia shows the fastest growth rate. The correlation between disease burden and SDI level was negligible. (3) The disease burden in males consistently exceeded that in females, with prevalence and YLDs rates rising sharply after age 60. (4) The main causes and their attributable proportions were: ischemic heart disease (34.53%), hypertensive heart disease (22.53%), other cardiomyopathies (7.61%), chronic obstructive pulmonary disease (6.51%), and congenital heart anomalies (5.69%), with their distribution patterns differing across age groups and regions. These findings provide insights for understanding and addressing the global HF burden.

Although a few studies have shown a declining trend in the global burden of HF in recent decades, more studies have confirmed an upward trend [13–15], which is consistent with our findings. Considering multiple factors in recent decades, including population aging, changes in the spectrum of underlying diseases, and advances in diagnostic and therapeutic technologies, the increasing trend in disease burden appears more reasonable and explanatory.

Population aging is a crucial factor contributing to the increased global burden of HF. Studies have shown that the incidence of HF increases significantly with age, with adults over 60 having about 20 times the risk of developing HF compared to younger individuals [24], which is consistent with the age distribution characteristics of HF disease burden revealed in this study. As age increases, various structural and functional changes occur in the cardiovascular system due to biological aging mechanisms such as decreased function of cellular protein quality control systems, mitochondrial dysfunction, and dysregulation of nutrient sensing [24]. These changes, including left ventricular hypertrophy, decreased left ventricular diastolic function, left atrial enlargement, atrial fibrillation, myocardial fibrosis, and cardiac amyloidosis, make older adults more susceptible to HF [25]. Moreover, older adults are often exposed to cardiovascular risk factors such as hypertension and diabetes for extended periods, which accumulate over time and further increase the risk of HF [25]. As the global population age structure changes and the proportion of elderly population continues to rise, it directly leads to an increase in the number of HF patients [25].

The rising prevalence of underlying diseases leading to HF is another key factor in the increasing burden of HF [15]. Consistent with the results of this study, ischemic heart disease remains one of the main causes of HF [17]. Research has found that over the past 30 years, the burden of ischemic heart disease has shown an increasing trend in the population aged 80 and above. With advancements in medical technology, the survival rate of patients with ischemic heart disease has improved, but this has also increased the overall burden of ischemic heart disease and HF [26]. Hypertensive heart disease (HHD) is currently the second major cause of HF. Despite substantial improvements in hypertension treatment and control in recent decades, the prevalence of HHD and its associated risk of HF continue to increase, possibly due to factors such as population aging, limitations of antihypertensive therapy, and myocardial interstitial fibrosis [27]. COPD and HF share many common risk factors, such as smoking, hypertension, and diabetes, while COPD-related systemic inflammation may promote the development of HFpEF. The similarity in symptoms between COPD and HF (such as dyspnea) also increases the difficulty of diagnosing HF in COPD patients, often leading to delayed diagnosis and affecting the progress in prevention and treatment of HF caused by COPD [28, 29].

Advancements in diagnostic and treatment technologies can affect the statistical values of HF disease burden. With the development of diagnostic techniques and the promotion of routine physical examinations, some early cases of HF that were previously difficult to detect can now be diagnosed, which may lead to an increase in the ASPR of HF in recent years [17]. The improvement in HF treatment methods has extended patients’ survival periods, and while this is a positive trend, it also means that more patients survive for longer periods, thereby increasing the overall disease burden [25].

Notably, This study revealed a potential turning point in the global burden of HF during 2019–2021. During this period, high-SDI regions showed a significant decline in ASPR and ASYR, while other SDI regions, although still increasing, showed a decelerated growth rate, ultimately leading to the observed decline in global burden from 2018 to 2021. These trends might appear to reflect recent significant advances in HF management, including the widespread adoption of innovative medications such as ARNI and SGLT2 inhibitors, the evolution of mechanical support devices, as well as continuous optimization of clinical guidelines. However, this period coincided with the global COVID-19 pandemic, which likely significantly influenced HF burden statistics through multiple pathways: First, changes in healthcare-seeking behavior during the pandemic, such as delayed medical visits and reduced outpatient follow-ups, may have led to underreporting of new cases; second, the diversion of medical resources toward COVID-19 treatment and limited access to specialized HF care may have affected timely disease detection and diagnosis; third, COVID-19 infection itself could exacerbate existing HF or trigger new cases, while pandemic-related lifestyle changes (such as reduced physical activity and dietary changes) and increased psychological stress may have adversely affected cardiovascular health; furthermore, changes in data collection systems and healthcare policies during the pandemic may have impacted the accuracy of statistical results [30, 31]. Therefore, whether the observed trends in disease burden during this period will persist requires further observation as the pandemic’s impact gradually subsides.

Nonetheless, from a purely data perspective, HF burden metrics since 2019 have indeed shown more positive trends compared to previous periods, offering encouraging signs for HF management and prevention efforts. While continuing to promote standardized care and optimize treatment strategies, future research should focus on monitoring global HF burden trends in the post-COVID era while establishing more robust disease surveillance systems to enhance data quality and reliability during exceptional circumstances.

Regarding the correlation between regional socioeconomic levels and HF burden, studies have shown that populations with lower socioeconomic status face a higher burden of HF. Individuals from lower socioeconomic classes have a 61% higher risk of developing HF compared to those from higher classes [14]. Some less developed regions and countries (such as West Sub-Saharan Africa, East Sub-Saharan Africa, South Asia, Southeast Asia, and Oceania) show an upward trend in HF burden [17]. Theoretically, uneven distribution of medical resources may result in HF patients in some regions being unable to receive timely and effective treatment, thereby increasing the overall disease burden [32]. However, the data analysis in this study only revealed a negligible correlation between regional SDI levels and HF disease burden. Even within the high SDI Western European region, France and Iceland have become the countries with the highest and lowest ASPR of HF globally, while Germany and Austria have become the countries with the fastest worsening and most significant improvement in HF prevalence burden, respectively. This marked regional difference is consistent with some recent research findings [33, 34]. Studies have also shown that even within the same developed country, there are significant regional differences in HF burden across different areas [35]. This suggests that when considering the impact of socioeconomic factors, it may be necessary to comprehensively consider various other factors, such as age structure, lifestyle, health policies, medical insurance systems, and so on [34].

In terms of representative regions, France and Sweden exhibit high ASPR, which may be attributed to their well-established healthcare systems, accurate diagnostic reporting systems, and high levels of population aging. Additionally, improvements in cardiovascular disease treatment have extended patient survival, consequently increasing the number of chronic HF patients. Germany shows a significant increase in disease burden, potentially linked to its distinctive healthcare service model (characterized by longer hospital stays and higher hospitalization rates), comprehensive electronic health record system, and region-specific risk factors (such as high prevalence of alcoholic cardiomyopathy) [34]. The marked increase in HF disease burden in South Asia likely results from multiple interacting factors: a large population base undergoing rapid aging, westernization of lifestyle leading to increased cardiovascular risk factors (particularly ischemic heart disease and diabetes), younger age at onset (approximately 10 years younger than Western Europe), and high comorbidity burden. Moreover, high out-of-pocket expenses in the healthcare system limit patient access to care, while poor adherence to treatment guidelines adversely affects disease outcomes [36–38].

The results of this study show that the disease burden of HF in males consistently exceeds that in females, especially after the age of 60, where this gender difference becomes more pronounced. This is consistent with some other recent research findings [39, 40]. It is worth noting that previous studies have shown that after the age of 85, the incidence of HF in females surpasses that in males [39], a pattern not observed in the current study. The potential mechanisms by which gender influences HF disease burden may involve factors such as the protective effect of estrogen in females, higher exposure to traditional risk factors like smoking and alcohol consumption in males, and differences in the intensity of various pathophysiological mechanisms between genders. For instance, enhanced sympathetic nervous activity, metabolic-related risk factors, and chronic inflammation may play a more important role in males [39, 41].

Although the global overall distribution of HF causes shows no significant differences across different years and genders, there are substantial variations across different age groups and regions. This provides important evidence for developing targeted prevention and intervention strategies. For instance, for children and adolescents, the focus should be on preventing and treating congenital heart anomalies; for young adults, other cardiomyopathies and rheumatic heart disease should be prioritized; for middle-aged and elderly populations, the focus should be on preventing and treating ischemic heart disease and hypertensive heart disease; for those over 70, the contribution of chronic obstructive pulmonary disease and non-rheumatic valvular disease should not be overlooked, particularly the co-occurrence or misdiagnosis of chronic obstructive pulmonary disease and HF. For most regions globally, especially Eastern Europe, the prevention and treatment of ischemic heart disease should be a priority; in sub-Saharan Africa and East Asia, particular attention should be paid to controlling hypertensive heart disease, while East Asia should also focus on the prevention and treatment of chronic obstructive pulmonary disease. These regional differences in causal distribution are consistent with the results of related studies by the GBD collaboration group [42].

This study has several limitations.(1)Primarily, the GBD study assumes a single etiology for each case of HF, despite the possibility of multifactorial or comorbid causes. This assumption facilitates practical estimations and elucidates the most prevalent and clinically relevant drivers of HF. Although this approach simplifies the complex pathophysiology of HF, the standardized attribution method enables systematic identification of predominant causes across different populations, thereby providing an essential foundation for evidence-based public health planning. As a result, it offers clear, actionable insights for healthcare systems to prioritize preventive interventions and optimize resource allocation. This pragmatic approach is particularly valuable for developing targeted population-level strategies, while still acknowledging the multifactorial nature of individual cases (2). The COVID-19 pandemic likely disrupted data collection, diagnosis, and treatment processes for rheumatic heart disease during 2020–2021. Although these disruptions posed significant challenges, the GBD study implemented various methodologies to maintain data quality, including modeling techniques and analytical adjustments, though these compensatory measures may introduce their own uncertainties. Future research should focus on monitoring HF burden trends in the post-COVID era to validate whether the turning point observed during 2019–2021 represents a sustainable shift(3). Despite its extensive coverage, the GBD database’s accuracy and completeness vary across regions, with potential inadequacies in data comprehensiveness and representativeness, particularly in low- and middle-income countries. Temporal and geographic variations in medical practices, diagnostic technologies, and reporting systems may introduce biases in long-term trend analyses. Nevertheless, the GBD study remains the most authoritative and systematic source of global disease burden data available. Our findings on HF burden trends align with previous related studies, and future analyses using alternative data sources may provide further validation of these observations(4). Lastly, the GBD database primarily provides population-level information, lacking detailed individual patient data. This limitation constrains our ability to conduct in-depth epidemiological analyses of specific subtypes or patient cohorts. However, this macro-level approach effectively supports our primary objective of understanding broad patterns and identifying public health priorities, particularly in highlighting regional disparities and temporal trends that can inform targeted interventions.

## Conclusion

The global burden of HF increased significantly over recent decades, with a potential turning point in 2019 and marked regional disparities. The findings underscore the importance of developing targeted prevention and control strategies. Particular attention should be paid to regions with heavy disease burdens or rapid growth rates, such as high-income North America and South Asia, to formulate HF prevention and control measures tailored to local conditions. It is essential to strengthen the management of ischemic heart disease and hypertensive heart disease while not neglecting other major causes such as cardiomyopathies, chronic obstructive pulmonary disease, and congenital heart anomalies.

In addition to promoting standardized care and optimizing treatment strategies of HF, future research should focus on monitoring HF burden trends in the post-COVID era to evaluate whether the turning point observed during 2019–2021 represents a sustainable shift. These findings will be instrumental in informing evidence-based health policy development and implementation.

## Electronic supplementary material

Below is the link to the electronic supplementary material.

Supplementary Material 1

## Data Availability

The comprehensive data from GBD 2021 is publicly available and can be downloaded at https://vizhub.healthdata.org/gbd-results/.

## Abbreviations

HF: Heart failure
YLDs: Years lived with disability
GBD: Global Burden of Diseases, Injuries and Risk Factors Study
EAPC: Estimated annual percentage change
SDI: Socio-demographic Index
UI: Uncertainty intervals
ARNI: Angiotensin Receptor-Neprilysin Inhibitor
SGLT2: Sodium-Glucose Cotransporter-2
sGC: Soluble Guanylate Cyclase
LVADs: Left ventricular assist devices
IHME: Institute for Health Metrics and Evaluation
HALE: Healthy life expectancy
COPD: Chronic obstructive pulmonary disease
ASPR: Age-standardized prevalence
ASYR: Age-standardized YLDs rate
ESC: European Society of Cardiology
DisMod-MR: Disease Modeling-Meta Regression tool
MR-BRT: Meta-regression—Bayesian, Regularized, Trimmed
