# Supplementary material for: Global, regional, and national burden of heart failure and its underlying causes, 1990–2021: results from the global burden of disease study 2021
Source: Biomark Res. 2025 Jan 23;13:16. doi: 10.1186/s40364-025-00728-8 (PMC11755835; doi:10.1186/s40364-025-00728-8)
Supplement: Supplementary file 1 — Supplementary Material 1 [file 40364_2025_728_MOESM1_ESM.pdf]

# SUPPLEMENTARY MATERIAL

**Supplement to “Global, regional, and national burden of heart failure and its underlying causes, 1990–2021: results from the Global Burden of Disease Study 2021”**

## CONTENTS


SUPPLEMENTARY MATERIAL ........ 1

Figure S1 ........ 2

Figure S2 ........ 3

Figure S3 ........ 4

Figure S4 ........ 5

Figure S5 ........ 6

Figure S6 ........ 7

Figure S7 ........ 8

Figure S8 ........ 9

Figure S9 ........ 10

Figure S10 ........ 11

Figure S11 ........ 12

Table S1 ........ 13

Table S2 ........ 16

# Figure S1

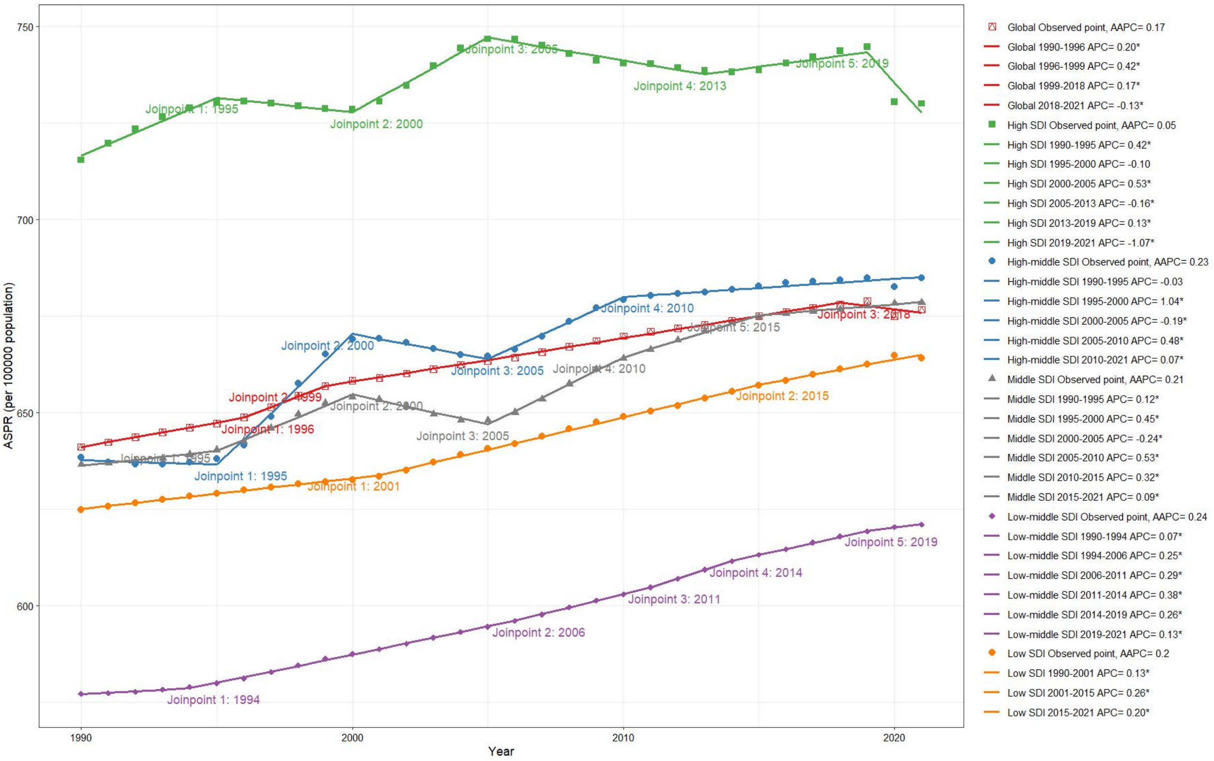


Figure S1: Temporal Trends in ASPR of HF by SDI Regions: A Joinpoint Regression Analysis, 1990-2021

Abbreviations: HF, heart failure; ASPR, age-standardized prevalence rate; SDI, socio-demographic index; APC, annual percent change; AAPC, average annual percent change. *P<0.05.

# Figure S2

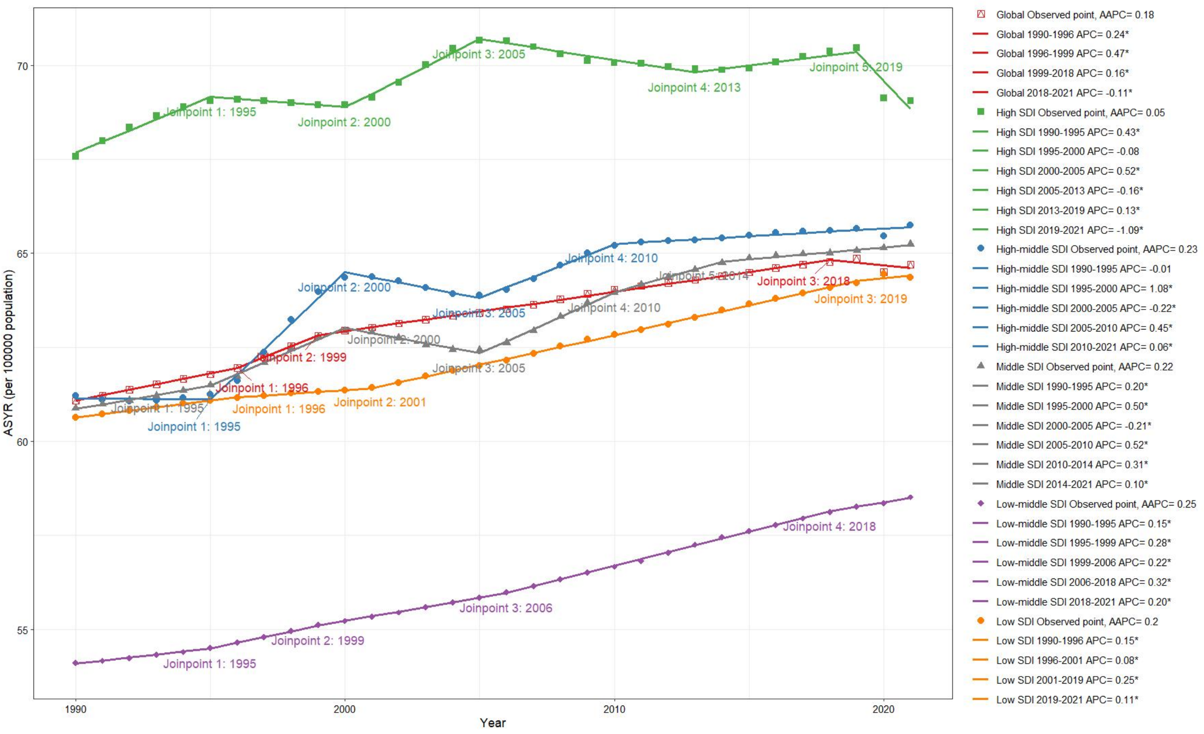


Figure S2: Temporal Trends in ASYR of HF by SDI Regions: A Joinpoint Regression Analysis, 1990-2021

Abbreviations: HF, heart failure; YLDs = years lived with disability; ASYR = age-standardized YLDs rate; SDI, socio-demographic index; APC, annual percent change; AAPC, average annual percent change. *P<0.05.

# Figure S3

A

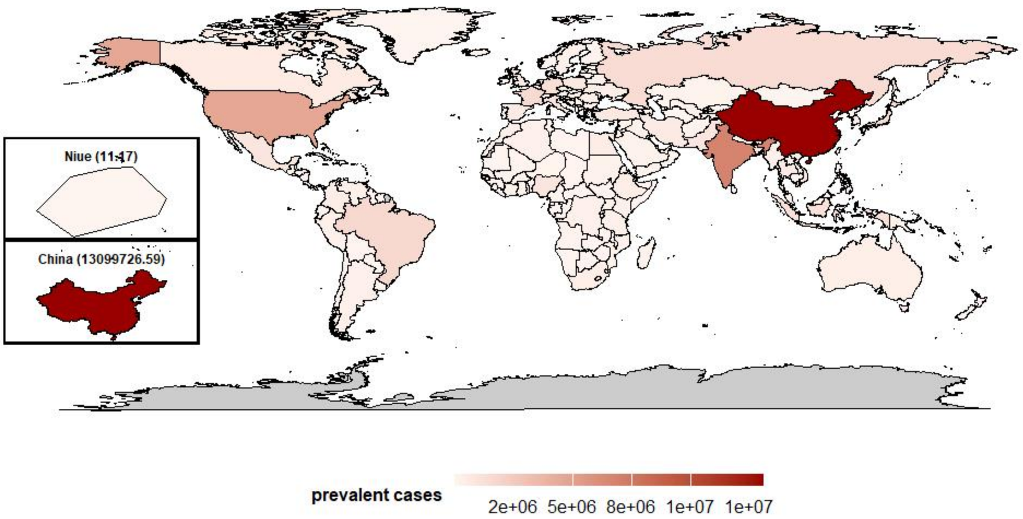


B

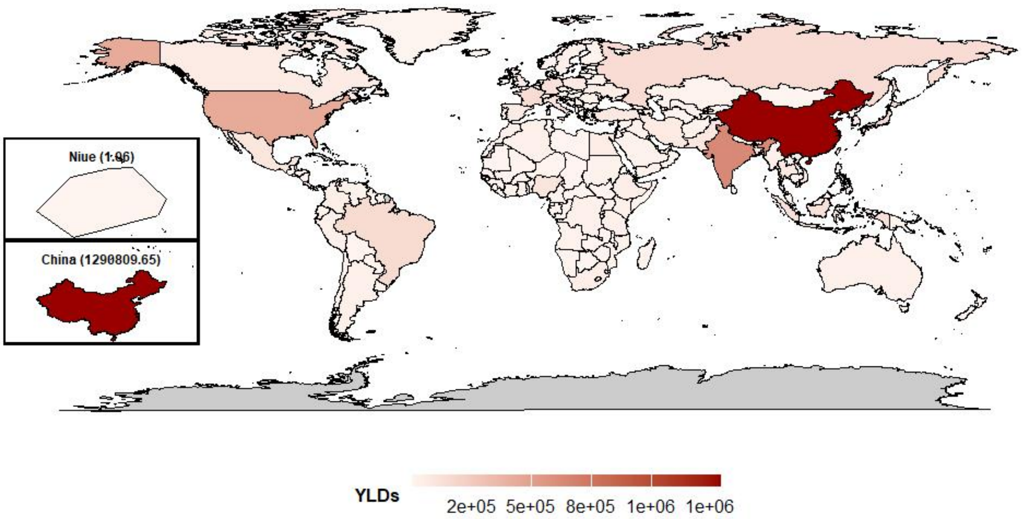


Figure S3: Geographic distributions of heart failure burdens across 204 countries and territories, for both sexes and all ages, 2021

(A) Prevalent cases of heart failure in 2021. Bottom-left small maps display the lowest (Niue) and highest (China) cases. (B) Years Lived with Disability (YLDs) of heart failure in 2021. Bottom-left small maps display the lowest (Niue) and highest (China) YLDs. Note: The legend uses scientific notation, where 'e' represents '10 to the power of'. For example, 2e+06 is equivalent to 2 × 10^6 or 2,000,000.

## Figure S4

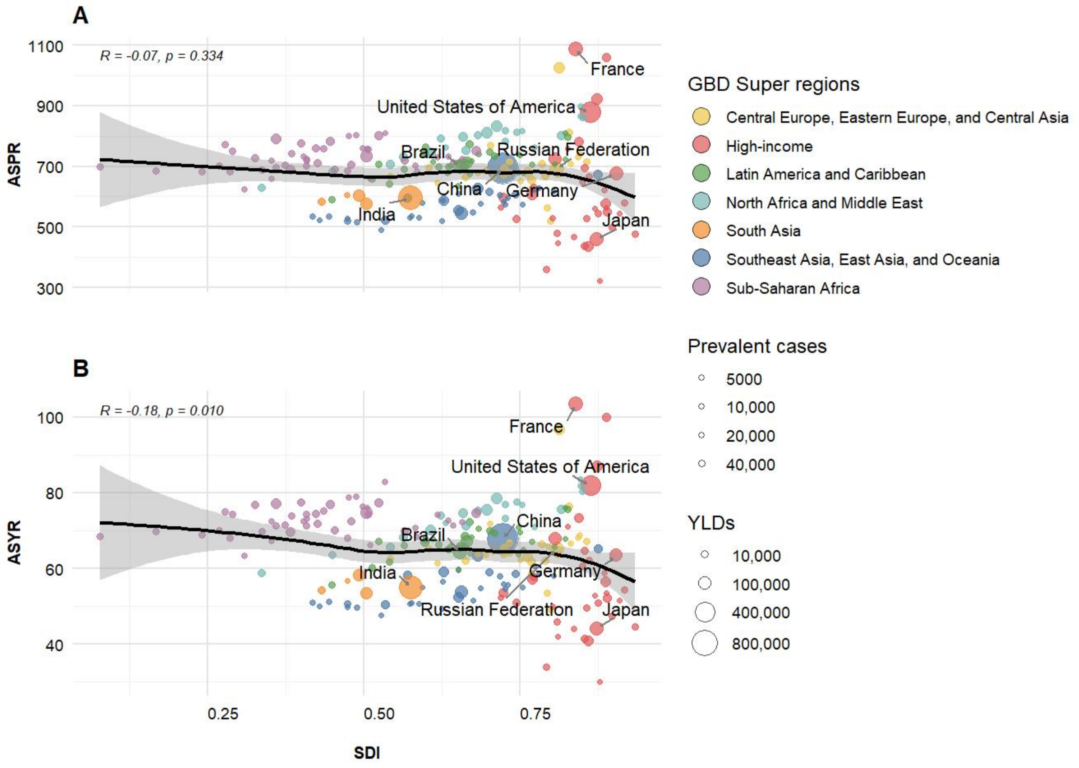


Figure S4: Burdens of heart failure in relation to SDI by 204 countries and territories, for both sexes, 2021

(A) ASPR of heart failure in relation to SDI. (B) ASYR of heart failure in relation to SDI. Note: Each point represents a country. Point size indicates absolute numbers of Prevalence or YLDs as per legend. Colors denote GBD super regions. Labeled countries represent those with the highest disease burden for each metric. Correlation statistics are based on Pearson's correlation test. Abbreviations: SDI = Socio-demographic Index; YLDs = Years Lived with Disability; ASYR = Age-standardized YLDs rate(per 100,000 population); ASPR = age-standardized Prevalence(per 100,000 population); GBD = Global Burden of Disease study.

# Figure S5

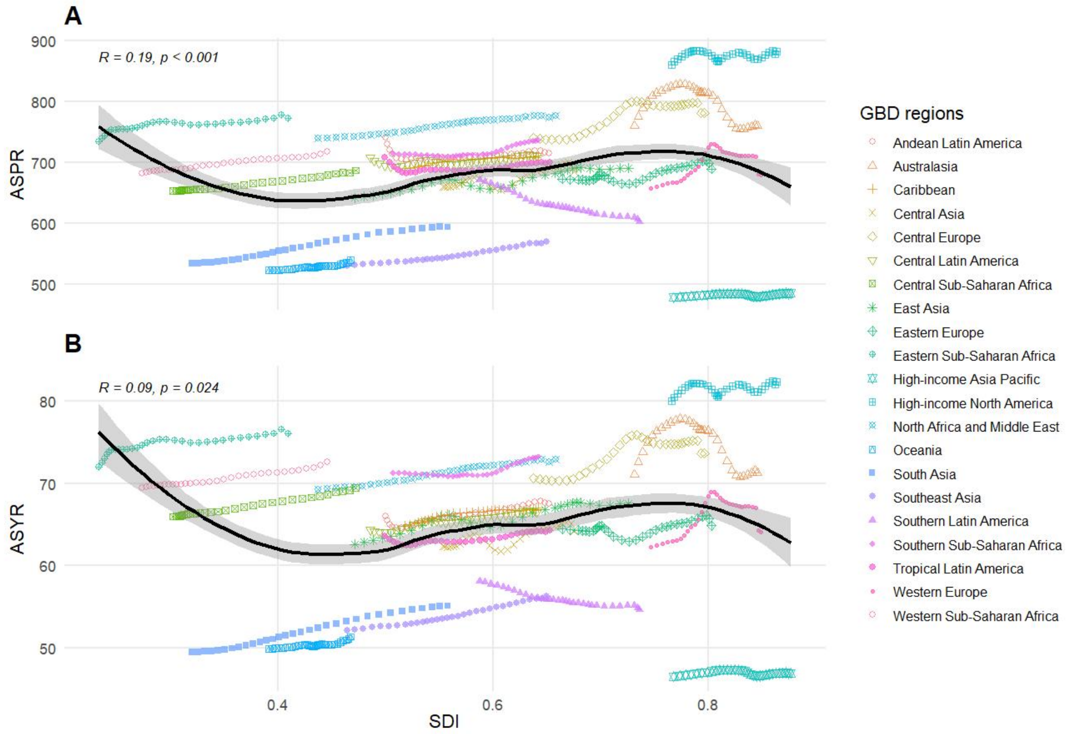


Figure S5: Burdens of heart failure in relation to SDI by 21 GBD regions, for both sexes, 2021

(A) ASPR of heart failure in relation to SDI. (B) ASYR of heart failure in relation to SDI. Note: Each point represents a region-year combination from 1990 to 2021. Colors/shapes denote different GBD regions as per the legend. Black lines represent the overall trend across all data points. R and p values indicate Pearson's correlation coefficients and their statistical significance, respectively. Abbreviations: SDI = Socio-demographic Index; ASPR = age-standardized Prevalence(per 100,000 population); YLDs = Years Lived with Disability; ASYR = age-standardized YLDs rate(per 100,000 population); GBD = Global Burden of Disease study.

# Figure S6

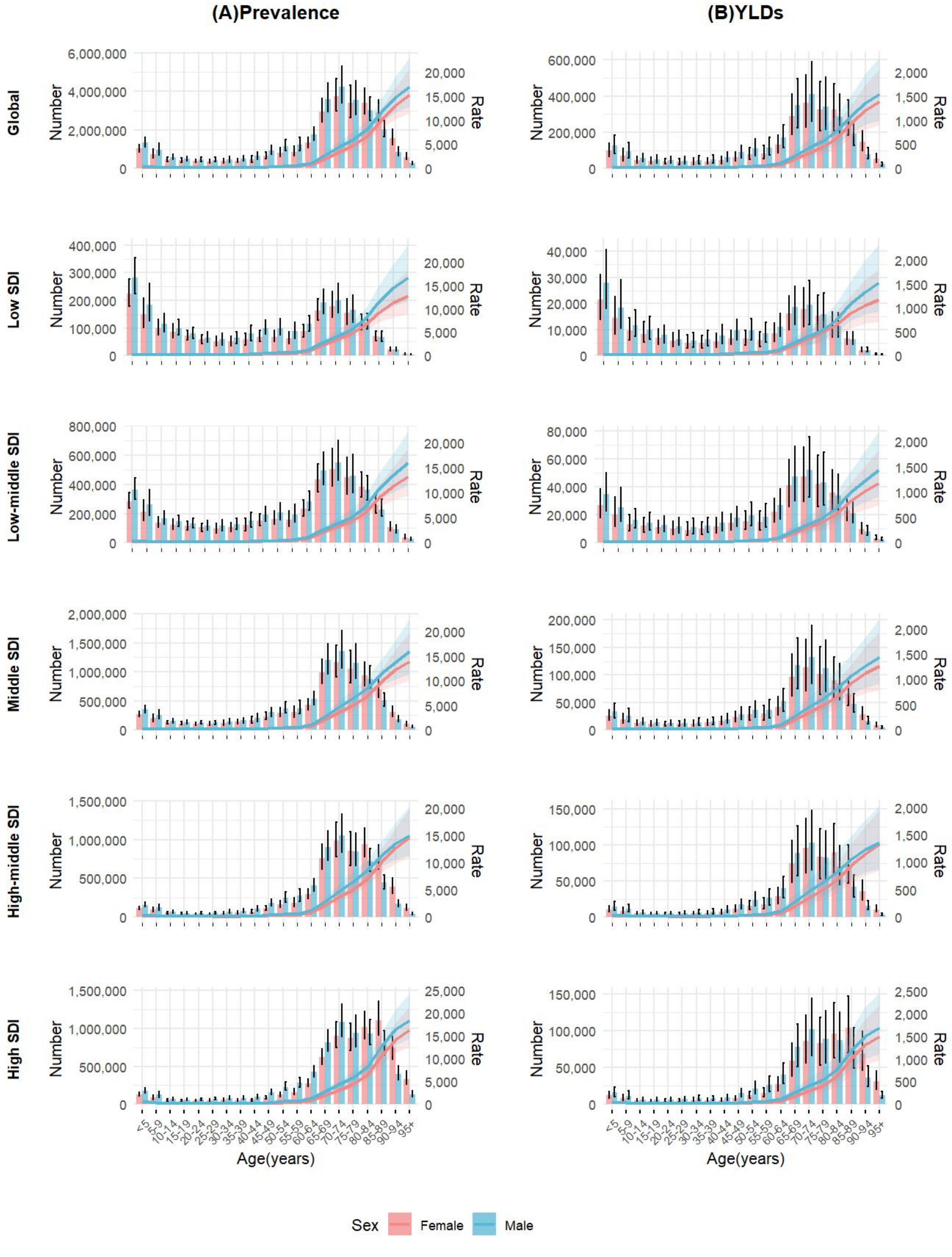


Figure S6: Global and Regional Trends of heart failure Burdens Across Age Groups by Sex, 2021

(A) prevalent cases and prevalence (per 100,000 population) of heart failure across different age groups by sex and SDI region. (B) YLDs and YLDs rate (per 100,000 population) of heart failure across different age groups by sex and SDI region. Note: Bars represent absolute numbers (left y-axis), while lines represent rates per 100,000 population (right y-axis). Vertical black lines indicate 95% uncertainty intervals. Each row represents a different SDI region, from top to bottom: Global, Low SDI, Low-middle SDI, Middle SDI, High-middle SDI, and High SDI. Abbreviations: YLDs = Years Lived with Disability; SDI = Socio-demographic Index.

## Figure S7

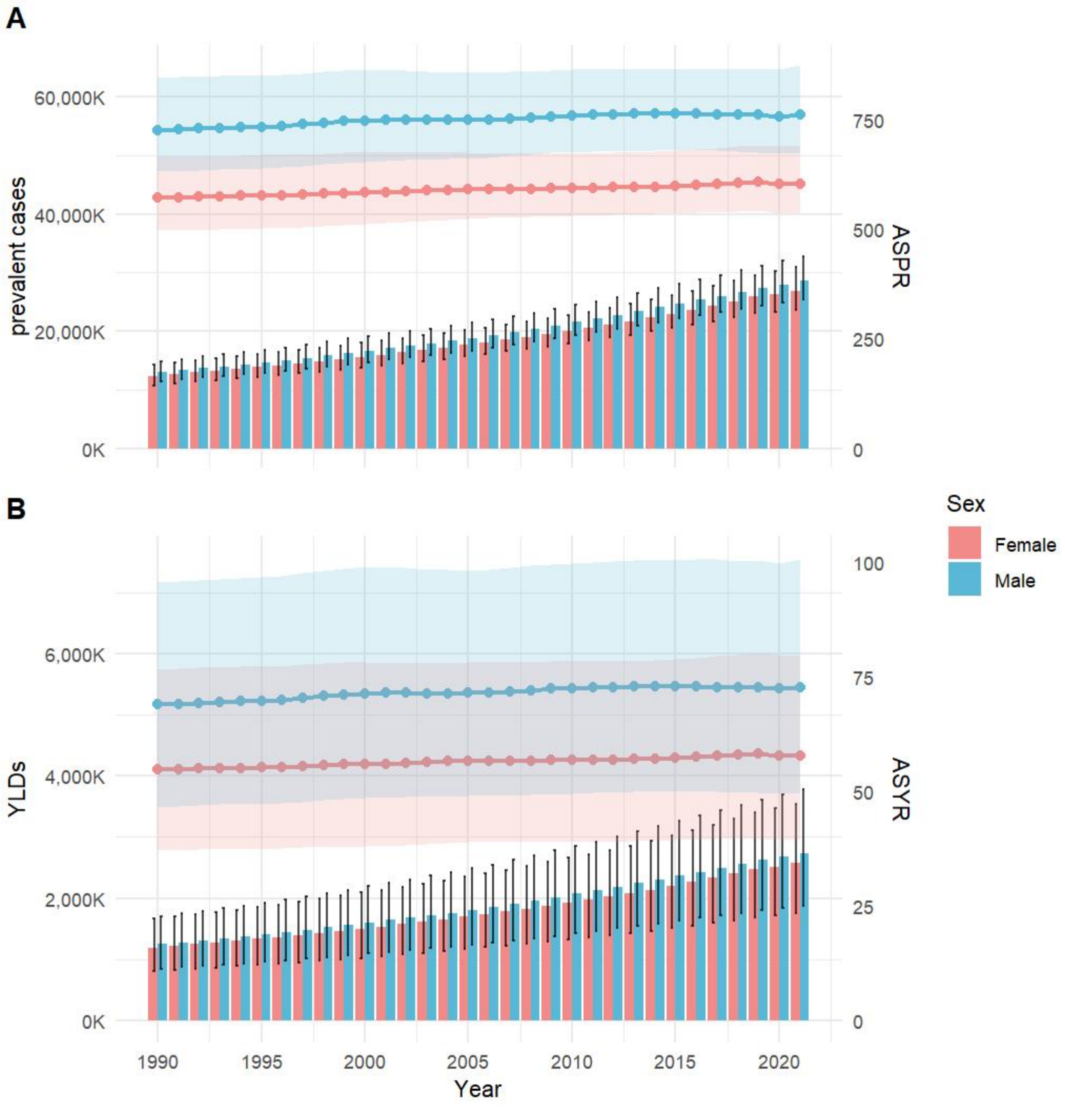


Figure S7: Global Trends of heart failure Burdens by Sex from 1990 to 2020

(A) prevalent cases and ASPR of heart failure by Sex from 1990 to 2020. (B) YLDs and ASYR of heart failure by Sex from 1990 to 2020. Note: Bars represent absolute numbers (left y-axis), while lines represent age-standardized rates per 100,000 population (right y-axis). Pink represents females, blue represents males. Vertical black lines indicate 95% uncertainty intervals. Abbreviations: ASPR = age-standardized Prevalence(per 100,000 population); YLDs = Years Lived with Disability; ASYR = Age-standardised YLDs rate(per 100,000 population).

# Figure S8

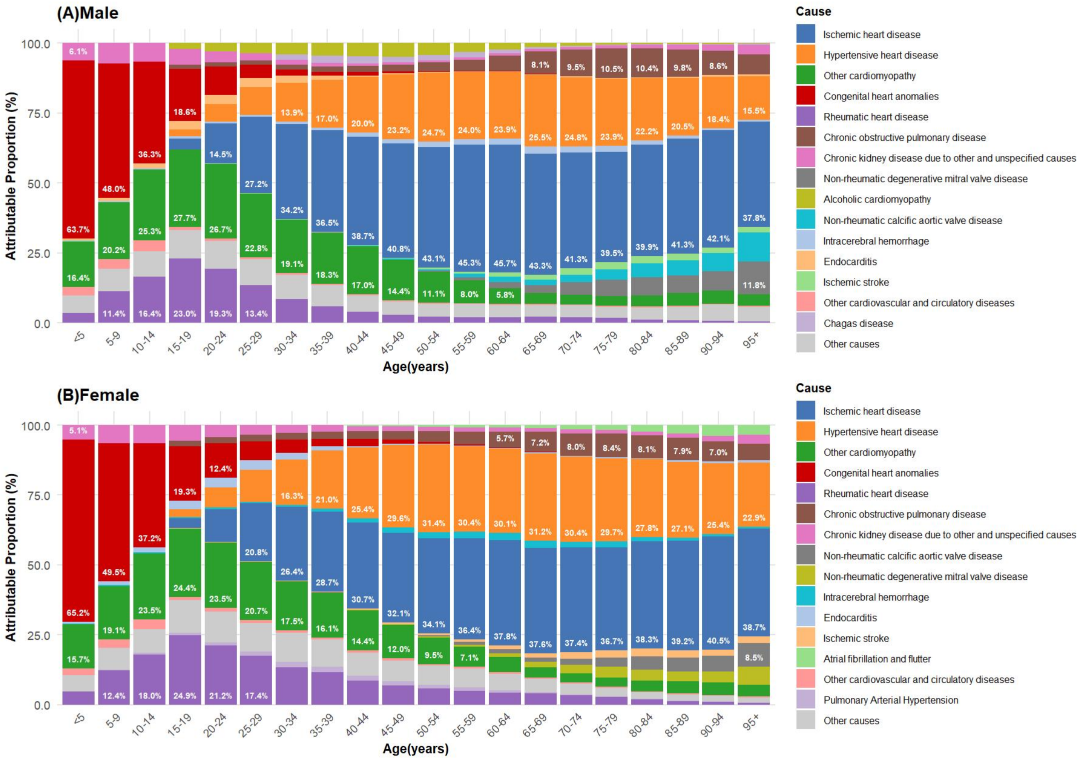


Figure S8: Proportion of heart failure prevalent cases attributable to major underlying causes by sex and age groups globally, 2021

# Figure S9

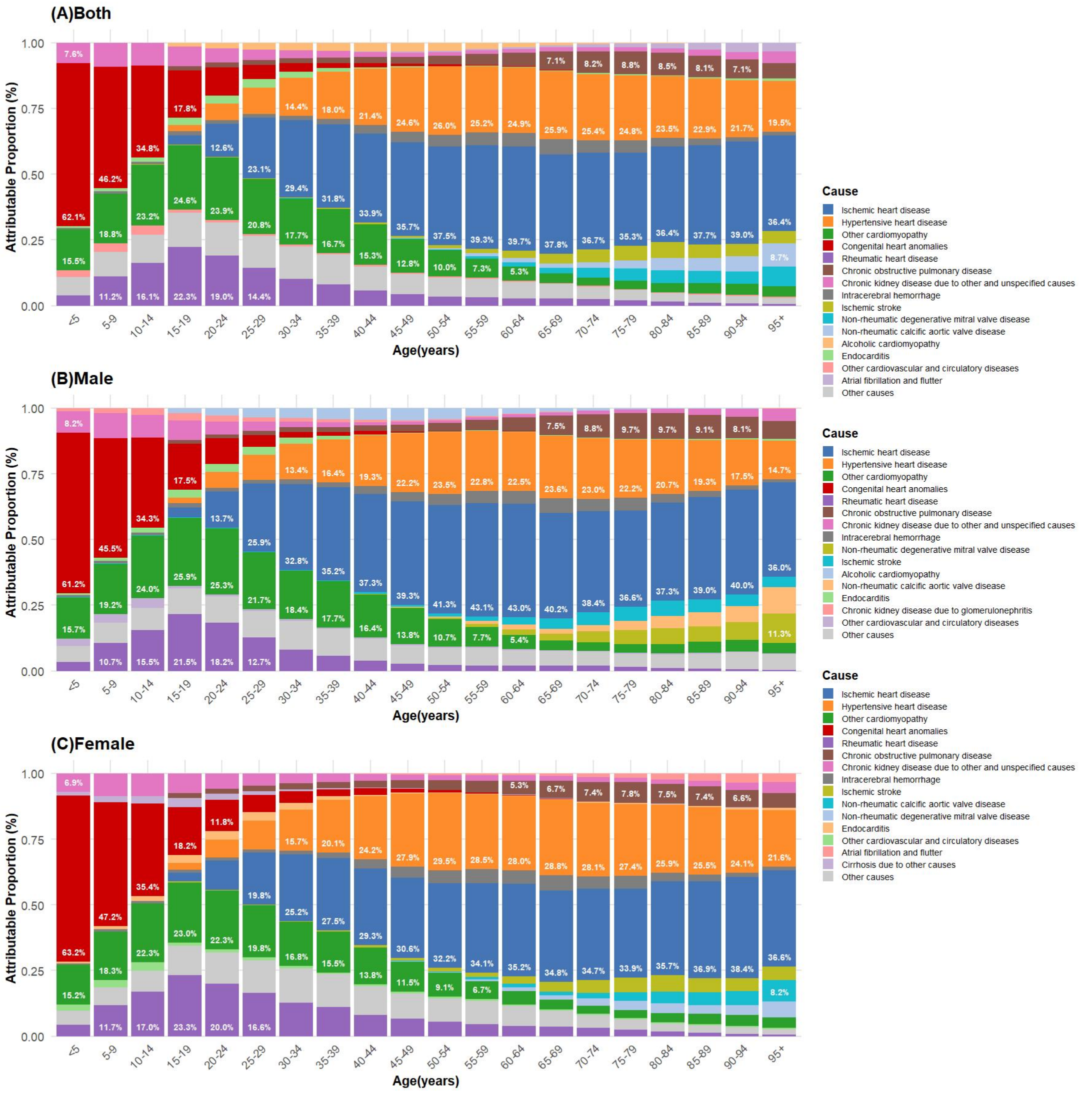


Figure S9: Proportion of YLDs attributable to major underlying causes by sex and age groups globally, 2021

Abbreviations: YLDs = years lived with disability

# Figure S10

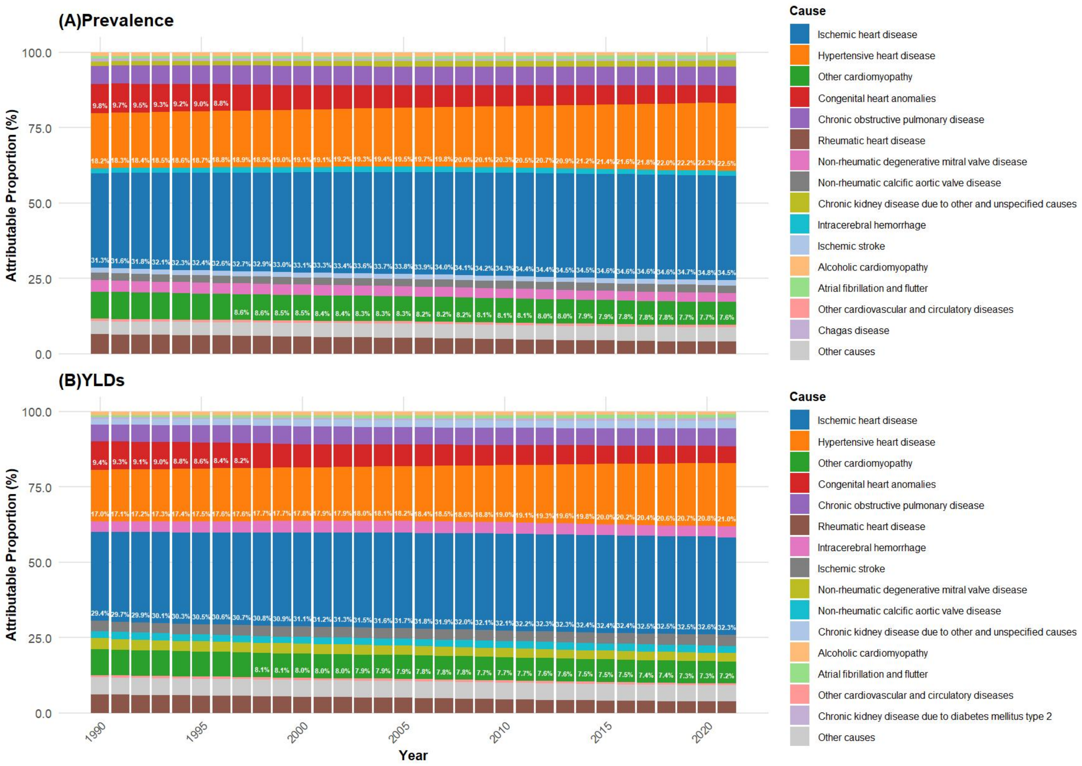


Figure S10: Proportion of heart failure burden attributable to major underlying causes from 1990 to 2020 globally, for both sexes and all ages

Abbreviations: YLDs = years lived with disability

# Figure S11

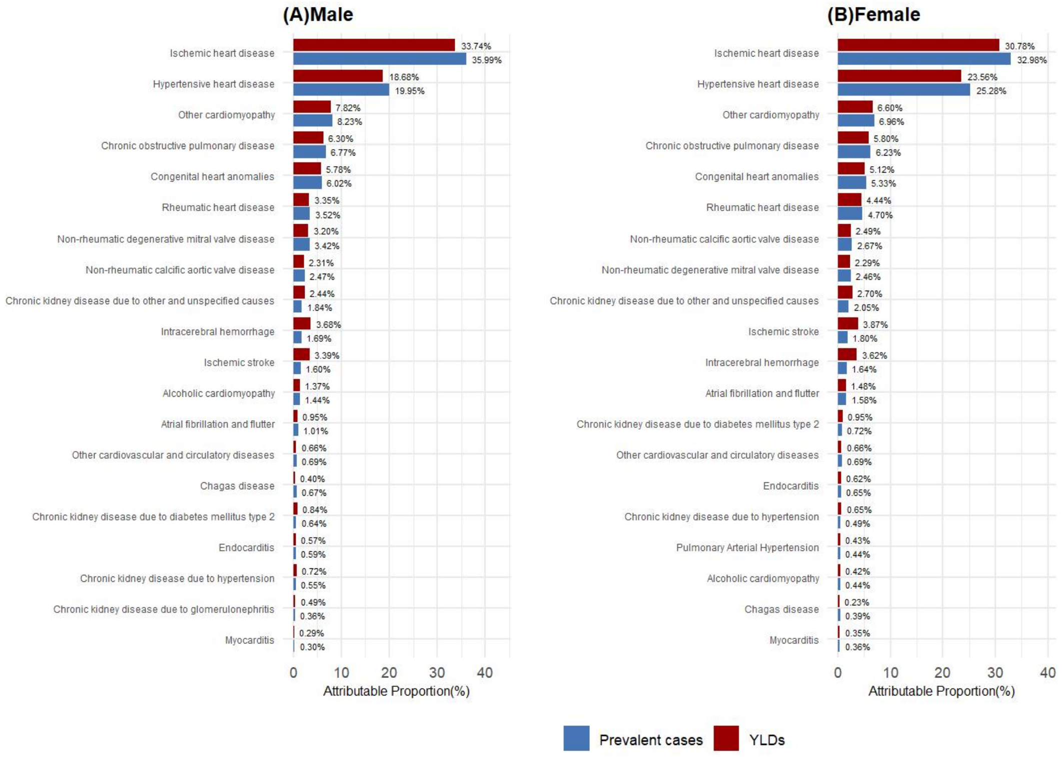


Figure S11: Proportion of heart failure prevalent cases and YLDs attributable to major underlying causes globally by Sex, for all ages, 2021

Abbreviations: YLDs = years lived with disability

# Table S1

Table S1 The prevalence burden of heart failure and its temporal trends by 204 countries and territories, for all ages and both sexes, 1990-2021

| | Prevalent cases (95% UI) | | ASPR (95% UI) | | |
|---|---|---|---|---|---|
| | 1990 | 2021 | 1990 | 2021 | 1990–2021 EAPC (95% CI) |
| **Central Asia** | | | | | |
| Armenia | 18745 (15788–22425) | 30353 (25728–36285) | 721.25 (591.42–875.25) | 769.96 (660.84–902.72) | 0.32 (0.26–0.39) |
| Azerbaijan | 33107 (28173–39184) | 58117 (49032–69397) | 653.75 (542.28–796.41) | 685.59 (577.86–828.31) | 0.22 (0.18–0.27) |
| Georgia | 40076 (33409–49617) | 39968 (33017–49476) | 707.12 (590.67–868.02) | 714.52 (604.95–864.68) | 0.08 (0.03–0.12) |
| Kazakhstan | 80992 (68476–97428) | 111376 (94373–134020) | 656.78 (545.46–809.9) | 694.78 (583.37–840.29) | 0.29 (0.24–0.34) |
| Kyrgyzstan | 21626 (18536–25954) | 32592 (27674–38597) | 675.87 (560.92–839.34) | 697.19 (581.54–843.99) | 0.16 (0.12–0.2) |
| Mongolia | 7864 (6744–9261) | 13717 (11706–16203) | 620.9 (518.98–769.35) | 645.43 (542.86–787) | 0.22 (0.17–0.26) |
| Tajikistan | 21361 (18161–25114) | 39081 (33588–45518) | 649.9 (532.35–793.54) | 666.05 (557.13–818.61) | 0.13 (0.08–0.17) |
| Turkmenistan | 14365 (12205–16691) | 25147 (21554–29559) | 658.47 (541.16–806.95) | 676.78 (569.83–817.86) | 0.15 (0.11–0.19) |
| Uzbekistan | 87985 (75680–103040) | 159100 (135661–187256) | 638.54 (530.28–794.02) | 663.55 (553.96–813.78) | 0.18 (0.14–0.23) |
| **Central Europe** | | | | | |
| Albania | 14673 (12563–17081) | 25637 (21009–31996) | 656.48 (546.82–788.62) | 664.37 (560.89–803.71) | 0.08 (0.06–0.1) |
| Bosnia and Herzegovina | 23884 (20061–28363) | 35558 (28957–43739) | 662.67 (557.54–797.95) | 671.69 (566.32–798.24) | 0.1 (0.07–0.12) |
| Bulgaria | 64422 (52440–80347) | 79115 (63645–100092) | 628.37 (528.4–759.35) | 626.81 (532.01–763.07) | -0.01 (-0.02–0) |
| Croatia | 29471 (24337–35791) | 39737 (33529–48151) | 575.06 (479.94–691.85) | 519.54 (448.92–606.08) | -0.57 (-0.76–-0.37) |
| Czechia | 98151 (79520–122021) | 155040 (125751–192217) | 789.03 (649.05–952.24) | 809.05 (685.65–975.53) | 0.6 (0.34–0.86) |
| Hungary | 83611 (68986–103301) | 115490 (93759–142400) | 649.04 (547.38–776) | 665.07 (566.96–787.8) | 0.07 (0.06–0.08) |
| Montenegro | 3980 (3336–4822) | 5609 (4556–6940) | 681.56 (571.57–829.29) | 677.95 (569.52–812.42) | 0.02 (0–0.04) |
| North Macedonia | 10997 (9280–13067) | 17398 (14001–21769) | 637.74 (536.69–764.67) | 648.95 (549.07–783.89) | 0.08 (0.07–0.09) |
| Poland | 374933 (328504–431527) | 673613 (624552–730848) | 934.6 (823.47–1064.87) | 1025.57 (957.48–1100.75) | 0.34 (0.24–0.45) |
| Romania | 157222 (129858–192923) | 220940 (180713–275453) | 644.02 (539.93–784.05) | 655.53 (557.85–788.18) | 0.34 (0.23–0.46) |
| Serbia | 51374 (42972–62315) | 81228 (66552–100851) | 547.29 (461.39–666.58) | 563.85 (477.16–670.58) | -0.38 (-0.58–-0.18) |
| Slovakia | 37028 (30917–44965) | 55371 (45201–69117) | 664.7 (563.96–803.22) | 673.39 (567.7–809.78) | 0.56 (0.39–0.72) |
| Slovenia | 15623 (12835–19161) | 28868 (23874–35778) | 702.38 (586.56–846.49) | 727.67 (623.7–871.85) | 0.18 (0.16–0.21) |
| **Eastern Europe** | | | | | |
| Belarus | 80043 (65044–97268) | 98771 (80936–121649) | 686.68 (565.86–827) | 707.58 (593.36–845.92) | 0.18 (0.13–0.22) |
| Estonia | 13195 (10902–16192) | 18476 (15227–23098) | 709.77 (598.81–855.78) | 738.48 (622.99–895.78) | 0.21 (0.18–0.24) |
| Latvia | 22555 (18323–28597) | 25909 (21142–32064) | 694.56 (573.75–853.92) | 707.93 (604.75–841.19) | 0.13 (0.09–0.16) |
| Lithuania | 29609 (24231–36518) | 37798 (30537–47062) | 707.18 (588.64–862.2) | 715.1 (602.41–857.38) | 0.08 (0.05–0.11) |
| Republic of Moldova | 26084 (21641–31519) | 35392 (29276–43583) | 659.36 (545.73–798.32) | 680.69 (584.91–816.33) | 0.16 (0.12–0.2) |
| Russian Federation | 1048726 (915634–1211154) | 1449979 (1264512–1692548) | 665.37 (584.67–758.06) | 690.56 (611.84–790.29) | 0.19 (0.15–0.23) |
| Ukraine | 428151 (365900–503617) | 458711 (395172–543139) | 686.59 (599.55–792.24) | 679.75 (596.33–782.71) | 0.03 (0–0.07) |
| **Australasia** | | | | | |
| Australia | 144777 (121150–178152) | 329434 (284109–388434) | 780.58 (661.66–950) | 781.35 (684.72–905.11) | -0.21 (-0.35–-0.08) |
| New Zealand | 24686 (21086–29276) | 49448 (43716–57133) | 659.62 (568.98–773.52) | 648.97 (577.88–735) | -0.2 (-0.29–-0.11) |
| **High-income Asia Pacific** | | | | | |
| Brunei Darussalam | 686 (598–782) | 1439 (1237–1685) | 439.6 (374.37–519.2) | 446.83 (384.65–525.32) | 0.04 (0.03–0.05) |
| Japan | 666093 (584110–768886) | 1404841 (1246526–1601283) | 472.61 (419.41–535.48) | 459.77 (422.32–503.18) | -0.13 (-0.15–-0.1) |
| Republic of Korea | 151903 (130701–175362) | 433738 (372555–505417) | 512.85 (439.07–601.37) | 576.44 (507.02–657.16) | 0.44 (0.32–0.57) |
| Singapore | 11083 (9611–12734) | 37719 (32117–45001) | 508.09 (433.41–589.72) | 529.3 (460.36–616.31) | 0.14 (0.13–0.14) |
| **High-income North America** | | | | | |
| Canada | 273024 (232426–322633) | 593733 (485413–721562) | 887.88 (762.35–1038.35) | 922.09 (779–1093.16) | 0.14 (0.03–0.25) |
| Greenland | 249 (214–290) | 417 (348–501) | 737.66 (610.22–873.27) | 757.37 (644.08–901.24) | 0.12 (0.11–0.14) |
| United States of America | 2605839 (2302375–2996268) | 4616875 (4290102–4976907) | 857.89 (764.58–973.38) | 877.27 (820.75–942.99) | -0.01 (-0.04–0.02) |
| **Southern Latin America** | | | | | |
| Argentina | 215098 (183457–254926) | 315729 (267550–373641) | 683.09 (586.4–809.52) | 595.06 (510.57–699.1) | -0.42 (-0.45–-0.39) |
| Chile | 70434 (60047–81923) | 146798 (124044–174737) | 667.46 (565.91–787.95) | 629.29 (538.46–736.29) | -0.12 (-0.15–-0.09) |
| Uruguay | 20854 (17564–24979) | 29305 (24346–35189) | 573.34 (488.97–675.25) | 564.22 (483.49–658.69) | -0.04 (-0.05–-0.03) |
| **Western Europe** | | | | | |
| Andorra | 279 (230–338) | 757 (617–927) | 556.32 (465.09–669.55) | 561.35 (475.71–665.2) | 0 (-0.02–0.01) |
| Austria | 105813 (87985–119972) | 121624 (104383–137200) | 933.17 (797.82–1042.92) | 694.44 (615.37–768.17) | -1.3 (-1.44–-1.16) |
| Belgium | 66932 (54722–83237) | 91808 (81989–108223) | 485.42 (410.52–583.73) | 435.4 (392–499.63) | 0.01 (-0.1–0.11) |
| Cyprus | 3219 (2665–3897) | 8411 (6891–10315) | 439.42 (371–525.02) | 465.59 (394.39–554.11) | -0.28 (-0.45–-0.1) |
| Denmark | 33608 (27708–41231) | 53563 (43756–66672) | 458.9 (388.68–547.76) | 498.93 (421.36–599.93) | 0.4 (0.17–0.62) |
| Finland | 41944 (34533–50065) | 78820 (62803–97021) | 651.76 (551.43–761.37) | 667.2 (560.12–788.78) | 0.12 (-0.09–0.33) |
| France | 832153 (681311–1027886) | 1505424 (1234606–1840699) | 1052.24 (881.14–1271.73) | 1085.7 (920.21–1284.19) | 0.01 (-0.11–0.14) |
| Germany | 729402 (601508–891774) | 1219790 (989142–1489906) | 642.98 (542.98–761.84) | 675.91 (574.85–799.7) | 0.97 (0.68–1.25) |
| Greece | 59375 (48597–72678) | 75336 (64320–89209) | 449.03 (376.8–532.01) | 359.14 (318.53–409.36) | -1.18 (-1.35–-1) |
| Iceland | 899 (751–1070) | 1678 (1374–2048) | 320.44 (268.75–376.57) | 322.43 (273.53–384.22) | -0.17 (-0.35–0.01) |
| Ireland | 20258 (16786–24448) | 39074 (32348–47555) | 519.55 (435.56–618.43) | 543.48 (460.97–649.95) | 0.15 (0.15–0.16) |
| Israel | 21937 (18257–26503) | 58945 (49094–71446) | 453.13 (378.74–547.68) | 478.23 (403.68–569.75) | 0.13 (0.06–0.21) |
| Italy | 549809 (479260–636523) | 1015901 (916641–1137019) | 703.57 (624.26–797.33) | 723.4 (661.02–796.44) | 0.25 (0.01–0.5) |
| Luxembourg | 2868 (2367–3541) | 5955 (4903–7290) | 595.9 (500.71–715.41) | 619.03 (526.62–745.52) | -0.1 (-0.45–0.26) |
| Malta | 1984 (1650–2429) | 4679 (3795–5782) | 515.43 (432.09–623.24) | 529.11 (450.23–627.36) | 0.55 (0.38–0.73) |
| Monaco | 334 (268–416) | 480 (384–597) | 531.01 (447.43–638.45) | 543.17 (466.55–643.76) | 0.05 (0.05–0.06) |
| Netherlands | 93206 (79296–111830) | 172714 (138771–211000) | 502.65 (435.33–589.98) | 548.9 (458.01–645.03) | 0.18 (0.08–0.28) |
| Norway | 36089 (31619–41846) | 53116 (46224–61799) | 565.52 (500.98–640.31) | 580.53 (519.04–661.13) | 0.1 (0.09–0.11) |
| Portugal | 59923 (48947–73957) | 119946 (97771–147918) | 490.95 (413.34–589.19) | 523.97 (446.43–624.87) | 0.07 (0.01–0.13) |
| San Marino | 180 (147–224) | 404 (329–507) | 557 (469.33–673.49) | 562.49 (476.55–682.09) | 0 (-0.01–0.02) |
| Spain | 265377 (213848–329168) | 558756 (503070–612934) | 538.55 (448.49–648.26) | 607.61 (549.73–664.94) | 0.07 (-0.03–0.18) |
| Sweden | 136679 (118569–159093) | 219066 (187827–258314) | 1026.82 (910.64–1160.91) | 1060.21 (936.14–1219.74) | -0.01 (-0.09–0.07) |
| Switzerland | 44620 (36248–55017) | 79865 (65124–98653) | 464.47 (388.13–556.71) | 477.03 (404.96–569.62) | 0.38 (0.1–0.67) |
| United Kingdom | 359495 (308145–427729) | 500717 (435455–583119) | 436.63 (379.96–504.56) | 436.47 (389.38–493.55) | -0.17 (-0.22–-0.11) |

TableS1(continued)

| | | | | | |
|---|---|---|---|---|---|
| **Andean Latin America** | | | | | |
| Bolivia (Plurinational State of) | 37109 (30096–46423) | 70160 (59358–83240) | 1005.64 (798.12–1265.05) | 765.32 (645.99–910.43) | -0.65 (-0.75–-0.56) |
| Ecuador | 44408 (38044–51050) | 113899 (96850–135561) | 709.13 (597.52–843.1) | 697.09 (590.96–837.76) | 0.04 (0–0.08) |
| Peru | 95222 (82643–109062) | 239199 (201461–282341) | 685.07 (582.4–811.74) | 709.27 (594.37–843.34) | 0.19 (0.17–0.21) |
| **Caribbean** | | | | | |
| Antigua and Barbuda | 427 (353–508) | 679 (561–826) | 726.07 (600.58–858.23) | 735.66 (619.48–882.51) | -0.02 (-0.06–0.02) |
| Bahamas | 1141 (974–1340) | 2489 (2081–3013) | 669.69 (560.46–795.89) | 706.84 (594.65–854.22) | 0.17 (0.16–0.18) |
| Barbados | 2077 (1708–2580) | 3347 (2770–4132) | 695.99 (585.12–842.04) | 740.14 (630.35–888.58) | 0.18 (0.17–0.19) |
| Belize | 829 (715–966) | 2145 (1832–2506) | 681.47 (575.17–825.67) | 715.1 (601.56–862.19) | 0.15 (0.15–0.16) |
| Bermuda | 428 (353–515) | 976 (791–1204) | 744.64 (620.23–887.56) | 810.64 (685.09–968.67) | 0.27 (0.25–0.29) |
| Cuba | 75035 (61777–91803) | 134801 (109120–163808) | 740.05 (611.44–900.81) | 771.68 (646.94–917.03) | 0.14 (0.13–0.14) |
| Dominica | 417 (351–502) | 502 (419–606) | 647.36 (539.53–783.75) | 678.97 (576.65–813.53) | 0.13 (0.12–0.14) |
| Dominican Republic | 28969 (24874–33588) | 69361 (58703–83822) | 672.92 (559.09–805.23) | 699.6 (589.97–850.3) | 0.1 (0.08–0.13) |
| Grenada | 540 (451–653) | 670 (562–817) | 638.28 (530.27–774.47) | 673.77 (574.41–813.19) | 0.12 (0.1–0.14) |
| Guyana | 2754 (2345–3222) | 3759 (3184–4514) | 604.25 (501.21–745.26) | 633.41 (529.89–764.58) | 0.13 (0.12–0.14) |
| Haiti | 19302 (16184–22634) | 42722 (36545–50129) | 553.16 (449.37–681.45) | 589.28 (493.53–716) | 0.2 (0.18–0.21) |
| Jamaica | 14536 (12353–17474) | 22420 (18681–27512) | 710.74 (596.82–861.74) | 740.26 (622.43–899.43) | 0.08 (0.05–0.12) |
| Puerto Rico | 26885 (22119–32340) | 54095 (43986–67695) | 749.51 (622.33–893.5) | 795.44 (666.26–962.81) | 0.2 (0.19–0.21) |
| Saint Kitts and Nevis | 253 (210–314) | 375 (312–452) | 625.8 (522.46–770.65) | 677.48 (571.26–808.86) | 0.25 (0.23–0.27) |
| Saint Lucia | 684 (574–810) | 1519 (1269–1852) | 671.1 (556.58–804.57) | 716.73 (606.05–861.36) | 0.2 (0.19–0.22) |
| Saint Vincent and the Grenadines | 542 (456–644) | 900 (739–1096) | 655.47 (540.81–795.79) | 701 (586.54–843.08) | 0.21 (0.2–0.21) |
| Suriname | 1778 (1499–2083) | 3915 (3271–4701) | 657.38 (545.55–789.73) | 677.08 (569.47–815.55) | 0.08 (0.07–0.09) |
| Trinidad and Tobago | 6072 (5109–7199) | 12129 (9932–14708) | 666.94 (556.41–808.86) | 705.6 (587.67–842.52) | 0.19 (0.18–0.2) |
| United States Virgin Islands | 551 (461–654) | 1165 (945–1459) | 680.29 (569.46–821.53) | 724.2 (614.06–867.03) | 0.17 (0.16–0.18) |
| **Central Latin America** | | | | | |
| Colombia | 135194 (116775–155292) | 375912 (315705–457086) | 671.91 (563.74–803.2) | 706.42 (598.72–853.71) | 0.19 (0.18–0.2) |
| Costa Rica | 15564 (13454–17943) | 38732 (32509–46528) | 741.29 (629.6–878.04) | 747.41 (632.96–887.34) | 0.07 (0.06–0.09) |
| El Salvador | 24134 (20882–27738) | 45087 (37828–54705) | 681.01 (577.14–807.43) | 691.73 (578.98–839.97) | 0.12 (0.1–0.14) |
| Guatemala | 28662 (24586–33293) | 74786 (63935–88169) | 652.76 (552.87–786.42) | 643.15 (545.44–773.02) | 0.04 (0.01–0.08) |
| Honduras | 17806 (15447–20364) | 44256 (37681–51988) | 672.1 (560.58–800.75) | 657.44 (551.11–788.78) | 0.01 (-0.02–0.03) |
| Mexico | 366966 (328042–407908) | 853851 (758489–969748) | 717.65 (636.15–814.22) | 712.68 (634.91–808.15) | 0.01 (-0.02–0.05) |
| Nicaragua | 15013 (13081–17111) | 35630 (30371–41783) | 710.1 (605.05–851.66) | 705.89 (597.59–841.38) | 0.05 (0.02–0.07) |
| Panama | 12187 (10388–14362) | 32356 (27691–38417) | 728.81 (608.02–883.98) | 733.93 (626–870.33) | 0.06 (0.04–0.07) |
| Venezuela (Bolivarian Republic of) | 87502 (75855–100532) | 207335 (174793–246713) | 762.46 (651.38–898.45) | 744.74 (631.2–885.39) | 0.02 (-0.01–0.04) |
| **Tropical Latin America** | | | | | |
| Brazil | 685835 (606232–767559) | 1633957 (1448187–1850611) | 707.61 (622.15–805.29) | 700.1 (620.96–789.68) | 0.04 (0.01–0.07) |
| Paraguay | 18951 (16256–21560) | 41055 (35144–48142) | 702.67 (592.52–827.65) | 698.21 (592.13–829.96) | 0.04 (0.02–0.07) |
| **North Africa and Middle East** | | | | | |
| Afghanistan | 43545 (36512–51688) | 94872 (82454–108120) | 623.74 (516.69–761.11) | 628.56 (527.04–759.26) | 0.03 (0.02–0.05) |
| Algeria | 115156 (98196–133677) | 282530 (240467–332317) | 768.09 (642.28–912.32) | 797.23 (678.17–959.48) | 0.14 (0.13–0.15) |
| Bahrain | 1932 (1678–2209) | 7969 (6691–9424) | 751.16 (633.17–903.24) | 814.75 (692.47–970.29) | 0.29 (0.28–0.3) |
| Egypt | 220848 (191953–253024) | 492104 (423228–569916) | 697.87 (581.02–828) | 744.85 (631.37–898.99) | 0.2 (0.19–0.21) |
| Iran (Islamic Republic of) | 254231 (229906–282073) | 600289 (533023–675048) | 774.54 (683.84–877.14) | 809.51 (717.15–921.19) | 0.13 (0.12–0.14) |
| Iraq | 80024 (69020–91700) | 198110 (170051–229127) | 723.18 (599.87–868.82) | 752.72 (640.94–906.68) | 0.15 (0.14–0.16) |
| Jordan | 15221 (13307–17374) | 65574 (56005–75716) | 777.02 (653.37–937.85) | 816.99 (695.06–962.64) | 0.18 (0.17–0.2) |
| Kuwait | 7378 (6409–8416) | 27741 (23425–32271) | 862.52 (721.11–1036.07) | 896.16 (753.32–1079.93) | 0.13 (0.1–0.16) |
| Lebanon | 17183 (14706–20264) | 48762 (40823–58604) | 769.58 (647.08–928.21) | 812.75 (685.34–966.24) | 0.19 (0.18–0.21) |
| Libya | 19325 (16817–22154) | 39796 (34166–46585) | 777.67 (655.26–924.49) | 782.54 (657.92–931.93) | 0.05 (0.03–0.07) |
| Morocco | 116759 (100121–135569) | 237333 (198818–283263) | 705.18 (585.55–842.85) | 731.94 (615.95–884.83) | 0.12 (0.12–0.12) |
| Oman | 7432 (6578–8376) | 20008 (17167–23303) | 692.49 (594.04–807.42) | 762.38 (641.21–921.08) | 0.31 (0.29–0.33) |
| Palestine | 9130 (7925–10504) | 23904 (20804–27495) | 729.86 (611.57–865.47) | 760.54 (641.97–905.18) | 0.12 (0.11–0.13) |
| Qatar | 1664 (1437–1918) | 12773 (10821–15135) | 801.89 (673.05–959.72) | 864.94 (734.12–1017.07) | 0.29 (0.27–0.31) |
| Saudi Arabia | 62838 (54831–71282) | 166148 (143791–193176) | 742.1 (621.9–893.67) | 802.96 (678.58–961.03) | 0.27 (0.26–0.28) |
| Sudan | 76840 (64619–88886) | 171230 (147765–196204) | 674.78 (545.79–814.43) | 706.62 (596.92–851.83) | 0.16 (0.15–0.16) |
| Syrian Arab Republic | 54080 (46645–61927) | 93998 (78984–113264) | 750.52 (620.4–893.95) | 791.02 (664.75–960.92) | 0.18 (0.15–0.22) |
| Tunisia | 43016 (36618–50468) | 97595 (82241–117308) | 775.23 (647.57–941.65) | 801.81 (674.93–959.44) | 0.1 (0.1–0.11) |
| Türkiye | 301041 (258654–350058) | 717157 (600899–859863) | 788.67 (665.15–942.32) | 831.38 (702.62–996.93) | 0.21 (0.18–0.23) |
| United Arab Emirates | 6774 (5906–7728) | 45606 (37436–54719) | 795.56 (676.99–937.9) | 861.26 (719.42–1024.69) | 0.26 (0.24–0.29) |
| Yemen | 46403 (40057–53629) | 125563 (108472–143824) | 657.75 (550.11–789.27) | 683.02 (579.74–818.08) | 0.14 (0.13–0.16) |
| **South Asia** | | | | | |
| Bangladesh | 336392 (291091–390595) | 809358 (685010–963194) | 540.54 (453.9–648.21) | 602.95 (510.28–719.33) | 0.38 (0.37–0.4) |
| Bhutan | 1736 (1494–1988) | 3674 (3127–4299) | 535.92 (452.04–639.88) | 604.69 (515.63–713.75) | 0.43 (0.41–0.44) |
| India | 2715763 (2448379–3016349) | 6722228 (5937217–7631762) | 532.4 (471.71–605.64) | 595.16 (526.69–677.85) | 0.42 (0.41–0.44) |
| Nepal | 57371 (49168–66047) | 134577 (112915–158904) | 519.93 (429.23–618.35) | 583.58 (487.67–696.96) | 0.4 (0.39–0.4) |
| Pakistan | 374279 (332523–416454) | 803962 (719443–893678) | 543.29 (479.67–620.59) | 577.37 (507.37–661.31) | 0.21 (0.2–0.23) |
| **East Asia** | | | | | |
| China | 4683303 (4036499–5435212) | 13099727 (11320895–15376467) | 644.58 (558.65–751.14) | 692.5 (607.26–802) | 0.23 (0.2–0.27) |
| Democratic People's Republic of Korea | 75073 (62108–91698) | 171489 (140046–214280) | 542.33 (440.94–677.83) | 595.34 (492.56–731.51) | 0.31 (0.29–0.32) |
| Taiwan (Province of China) | 89537 (73424–109883) | 263229 (213899–327993) | 625.66 (511.59–783.68) | 672.69 (558.99–823.74) | 0.24 (0.14–0.34) |
| **Oceania** | | | | | |
| American Samoa | 143 (123–165) | 243 (202–291) | 561.39 (464.19–683.8) | 582.34 (487–703.97) | 0.1 (0.09–0.11) |
| Cook Islands | 74 (62–88) | 149 (123–185) | 589.31 (479.78–724.57) | 629.67 (528.69–767.12) | 0.21 (0.21–0.21) |
| Fiji | 2028 (1722–2370) | 3654 (3042–4378) | 528.63 (435.8–645.22) | 547.66 (459.28–667.43) | 0.08 (0.07–0.09) |
| Guam | 451 (384–530) | 1242 (1021–1525) | 600.71 (496.67–735.5) | 616.53 (517.91–750.57) | 0.09 (0.07–0.1) |
| Kiribati | 183 (155–213) | 327 (277–384) | 472.75 (386.94–582.31) | 491.29 (408.82–597.63) | 0.11 (0.1–0.11) |
| Marshall Islands | 110 (94–128) | 163 (139–193) | 503.62 (420.37–623.99) | 531.65 (441.9–657.76) | 0.18 (0.17–0.19) |
| Micronesia (Federated States of) | 292 (246–343) | 337 (285–399) | 503.2 (416.94–619.1) | 530.85 (441.82–642.77) | 0.16 (0.15–0.17) |

TableS1(continued)

| | | | | | |
|---|---|---|---|---|---|
| Nauru | 25 (21–29) | 30 (26–36) | 511.06 (417.7–634.11) | 520.27 (433.88–634.68) | 0.05 (0.01–0.09) |
| Niue | 13 (11–16) | 11 (9–14) | 542.27 (447.95–664.44) | 573.55 (479.31–704.95) | 0.16 (0.15–0.16) |
| Northern Mariana Islands | 115 (99–131) | 252 (212–304) | 606.85 (498.52–738.44) | 623.15 (521.72–749.39) | 0.04 (0.02–0.06) |
| Palau | 52 (44–63) | 97 (80–120) | 543.1 (443.81–671.52) | 574.81 (480.41–706.22) | 0.15 (0.14–0.17) |
| Papua New Guinea | 9679 (8266–11305) | 26968 (23004–31318) | 516.17 (427.52–631.93) | 533.99 (449.15–646.06) | 0.08 (0.07–0.09) |
| Samoa | 534 (456–626) | 847 (725–1011) | 554.06 (456.73–678.06) | 578.18 (484.9–709.87) | 0.11 (0.1–0.12) |
| Solomon Islands | 807 (691–932) | 1876 (1608–2171) | 507.49 (417.56–630.58) | 522.92 (436.89–635.65) | 0.06 (0.05–0.08) |
| Tokelau | 7 (6–9) | 8 (7–10) | 545.44 (453.47–677.11) | 578.04 (481.18–706.97) | 0.17 (0.16–0.18) |
| Tonga | 347 (296–408) | 497 (419–591) | 569.96 (469.71–693.32) | 587.01 (489.95–711.26) | 0.07 (0.07–0.08) |
| Tuvalu | 31 (26–37) | 51 (43–62) | 499.61 (407.52–610.66) | 534.8 (449.39–652.75) | 0.2 (0.19–0.21) |
| Vanuatu | 379 (321–442) | 919 (785–1079) | 516.36 (425.14–631.16) | 527.04 (438.44–637.51) | 0.05 (0.05–0.06) |
| **Southeast Asia** | | | | | |
| Cambodia | 24706 (20940–28769) | 58857 (49295–70911) | 480.76 (391.12–599.51) | 520.24 (430.79–644.82) | 0.27 (0.25–0.29) |
| Indonesia | 506390 (450318–568265) | 1099460 (963117–1264038) | 512.47 (445.76–591.2) | 543.69 (475.8–623.55) | 0.17 (0.16–0.19) |
| Lao People's Democratic Republic | 10363 (8714–12175) | 22534 (19057–26892) | 473.84 (389.84–585.4) | 513.59 (425.69–630.29) | 0.27 (0.25–0.29) |
| Malaysia | 60614 (52350–69864) | 157561 (132837–187768) | 562.22 (467.68–680.71) | 607.56 (512.28–729.19) | 0.26 (0.25–0.27) |
| Maldives | 594 (512–686) | 1975 (1698–2308) | 556.77 (463.16–683.24) | 610.49 (513.09–738.67) | 0.31 (0.3–0.33) |
| Mauritius | 4084 (3473–4888) | 9549 (7781–11679) | 568.03 (470.28–685.29) | 604.18 (502.21–729.63) | 0.17 (0.16–0.18) |
| Myanmar | 109861 (91666–131882) | 229237 (191128–279783) | 488.89 (403–600.89) | 523.75 (431.84–642.52) | 0.25 (0.23–0.26) |
| Philippines | 180441 (161393–202033) | 429779 (382305–488255) | 541.36 (472.74–624.76) | 554.2 (488.33–636.83) | 0.06 (0.05–0.07) |
| Seychelles | 340 (286–408) | 598 (501–722) | 556.34 (463.23–673.02) | 594.98 (498.36–716.76) | 0.21 (0.2–0.22) |
| Sri Lanka | 61489 (52410–72628) | 148885 (124476–184153) | 569.7 (471.53–690.28) | 613.63 (515.76–742.3) | 0.24 (0.23–0.26) |
| Thailand | 198340 (167076–235407) | 608167 (495442–751305) | 577.62 (471.84–702.42) | 625.22 (524.14–761.67) | 0.25 (0.25–0.26) |
| Timor-Leste | 1670 (1439–1915) | 4782 (4054–5714) | 505.35 (417.54–620.91) | 533.05 (445.12–657.9) | 0.18 (0.17–0.19) |
| Viet Nam | 235316 (199947–280329) | 513272 (430942–627684) | 533.71 (440.32–655.49) | 586.76 (487.87–718.85) | 0.3 (0.29–0.31) |
| **Central Sub-Saharan Africa** | | | | | |
| Angola | 30756 (26160–35826) | 104717 (89723–120843) | 649.24 (537.21–790.68) | 689.2 (573.42–828.3) | 0.2 (0.19–0.22) |
| Central African Republic | 7686 (6530–8927) | 15685 (13338–18105) | 597.23 (502.36–734.55) | 622.89 (523.4–761.35) | 0.13 (0.13–0.14) |
| Congo | 8119 (6928–9435) | 20413 (17392–23728) | 646.09 (537.71–786.42) | 701.99 (593.22–845.67) | 0.28 (0.26–0.3) |
| Democratic Republic of the Congo | 121672 (103945–141943) | 303504 (261015–353103) | 657.72 (539.21–793.78) | 687.15 (573.81–838.12) | 0.14 (0.11–0.16) |
| Equatorial Guinea | 1433 (1227–1676) | 4936 (4245–5675) | 645.89 (533.58–776.35) | 722.77 (609.26–879.24) | 0.42 (0.4–0.45) |
| Gabon | 4359 (3681–5165) | 8075 (6916–9521) | 679.64 (568.22–819.17) | 730.51 (614.62–885.41) | 0.24 (0.23–0.25) |
| **Eastern Sub-Saharan Africa** | | | | | |
| Burundi | 20189 (17104–23952) | 45788 (39460–52975) | 709.55 (579.51–865.7) | 749.88 (628.32–908.12) | 0.18 (0.17–0.19) |
| Comoros | 1837 (1561–2158) | 4012 (3418–4708) | 769.09 (630.44–930.51) | 799.17 (671.77–959.74) | 0.11 (0.1–0.13) |
| Djibouti | 1365 (1171–1565) | 5440 (4677–6350) | 764.24 (627.21–928.74) | 802.11 (671.39–965.04) | 0.15 (0.13–0.17) |
| Eritrea | 10031 (8650–11519) | 23542 (20220–27217) | 696.54 (580.4–856.38) | 730.68 (614.13–887.12) | 0.13 (0.11–0.14) |
| Ethiopia | 172786 (153559–193722) | 434236 (385261–484800) | 717.64 (623.87–826.73) | 791.88 (691.7–919.32) | 0.31 (0.3–0.32) |
| Kenya | 90526 (80972–100756) | 215835 (193000–240565) | 787.08 (689.69–901.48) | 801.74 (706.67–918.3) | -0.39 (-0.55–-0.23) |
| Madagascar | 45670 (38834–53096) | 103465 (89248–119948) | 752 (624.46–912.4) | 774.23 (641.38–951.55) | 0.07 (0.06–0.08) |
| Malawi | 33130 (28271–38352) | 68387 (58916–79690) | 710.8 (585.23–857.74) | 743.39 (622.97–895.11) | 0.11 (0.09–0.13) |
| Mozambique | 48118 (40994–56213) | 104040 (89305–121427) | 703.01 (579.07–852.01) | 727.19 (597.43–883.63) | 0.06 (0.05–0.08) |
| Rwanda | 24627 (20815–28906) | 53608 (45321–63361) | 711.48 (583.6–865.63) | 757.36 (628.84–928.62) | 0.2 (0.19–0.22) |
| Somalia | 22108 (19088–25336) | 60944 (52370–70089) | 691.75 (576.84–845.46) | 699.75 (579.82–851.57) | 0.01 (0–0.02) |
| South Sudan | 22779 (19502–26761) | 35385 (30491–40896) | 744.26 (617.53–908.99) | 770.88 (646.07–928.48) | 0.1 (0.09–0.1) |
| Uganda | 60984 (52300–70864) | 151152 (129502–175167) | 728.9 (604.67–881.31) | 758.14 (624.83–922.41) | 0.11 (0.1–0.13) |
| United Republic of Tanzania | 99251 (84228–116496) | 241417 (206890–279163) | 751.41 (619.33–912.66) | 783.06 (655.3–940.15) | 0.33 (0.25–0.41) |
| Zambia | 26083 (22290–30278) | 67047 (57951–77171) | 718.5 (594.29–886.05) | 754.87 (632.48–909.43) | 0.13 (0.11–0.16) |
| **Southern Sub-Saharan Africa** | | | | | |
| Botswana | 4713 (4036–5551) | 10576 (9088–12402) | 680.94 (570.85–830.88) | 711.58 (594.71–853.38) | 0.13 (0.1–0.16) |
| Eswatini | 2598 (2224–3008) | 4251 (3620–4994) | 656.84 (543.32–800.74) | 681.67 (568.95–835.8) | 0.11 (0.08–0.14) |
| Lesotho | 6198 (5248–7342) | 7397 (6272–8748) | 639.34 (533.56–776.52) | 657.19 (552.81–796.39) | 0.07 (0.04–0.1) |
| Namibia | 5384 (4625–6293) | 10792 (9226–12626) | 697.43 (578.37–841.76) | 729.24 (612.08–877.39) | 0.14 (0.12–0.16) |
| South Africa | 169981 (151116–191718) | 332364 (294880–375401) | 726.24 (634.24–830.93) | 749.82 (665.03–852.66) | 0.13 (0.1–0.16) |
| Zimbabwe | 36465 (31010–42476) | 55998 (47752–64402) | 681.11 (561.12–828.09) | 684.67 (571.73–826.78) | -0.04 (-0.07–-0.01) |
| **Western Sub-Saharan Africa** | | | | | |
| Benin | 17862 (15227–20952) | 48182 (41398–55238) | 673.86 (561.25–820.03) | 705.53 (589.26–850.61) | 0.14 (0.13–0.14) |
| Burkina Faso | 33547 (28475–39595) | 80048 (69323–93161) | 656.11 (546.02–799.74) | 683.91 (569.14–834.84) | 0.14 (0.13–0.15) |
| Cabo Verde | 2147 (1834–2534) | 3603 (3059–4237) | 764.11 (643.22–921.11) | 811.49 (682.4–970.55) | 0.21 (0.19–0.22) |
| Cameroon | 36130 (30875–42231) | 110737 (95664–129381) | 666.67 (554.33–815.57) | 704.1 (590.86–860.6) | 0.16 (0.14–0.18) |
| Chad | 22380 (19013–26484) | 55583 (48125–64020) | 648.58 (539.18–795.97) | 681.67 (568.39–832.45) | 0.16 (0.15–0.18) |
| Côte d'Ivoire | 36346 (31496–41876) | 97588 (83781–113070) | 669.61 (549.91–817.58) | 708.63 (599.3–863.92) | 0.19 (0.17–0.21) |
| Gambia | 3340 (2865–3850) | 9106 (7843–10476) | 686.44 (569.31–827.44) | 717.83 (598.87–865.58) | 0.14 (0.13–0.15) |
| Ghana | 53258 (45449–61406) | 137281 (117985–161017) | 683.99 (562.23–824.68) | 718.01 (596.18–873.81) | 0.13 (0.12–0.14) |
| Guinea | 24591 (20916–29373) | 48454 (41935–55863) | 657.7 (543.13–811.52) | 687.58 (576.02–829.46) | 0.13 (0.12–0.14) |
| Guinea-Bissau | 3084 (2650–3616) | 6165 (5345–7052) | 626.16 (515.48–761.5) | 657.6 (555.46–792.3) | 0.16 (0.15–0.17) |
| Liberia | 9377 (8013–10980) | 18937 (16364–21799) | 675.13 (560.18–824.89) | 717.41 (601.33–864.68) | 0.21 (0.2–0.21) |
| Mali | 29967 (25593–35235) | 80152 (68905–93183) | 660.36 (548.16–798.97) | 700.37 (585.75–848.61) | 0.18 (0.17–0.19) |
| Mauritania | 8574 (7348–10037) | 19635 (16942–22906) | 700.17 (584.57–843.18) | 757.41 (635.8–918.67) | 0.26 (0.25–0.27) |
| Niger | 23822 (20542–27475) | 77428 (67323–89776) | 655.73 (546.11–802.03) | 684.97 (571.97–831.32) | 0.15 (0.14–0.17) |
| Nigeria | 361171 (321773–403574) | 851401 (758670–947878) | 696.19 (609.13–794.96) | 733.28 (645.9–841.95) | 0.16 (0.15–0.17) |
| Sao Tome and Principe | 554 (476–648) | 958 (819–1111) | 707.17 (595.53–849.07) | 759.89 (630.05–910.26) | 0.23 (0.22–0.23) |
| Senegal | 28724 (24546–33435) | 66088 (57357–76570) | 691.45 (573.22–835.49) | 724.6 (612.13–868.16) | 0.13 (0.12–0.14) |
| Sierra Leone | 16061 (13591–18894) | 31960 (27543–36904) | 663.05 (548.33–808.26) | 696.16 (580.85–837.14) | 0.14 (0.13–0.16) |
| Togo | 11513 (9899–13285) | 30616 (26514–35269) | 670.05 (555.78–813.89) | 700.37 (588.56–847.75) | 0.13 (0.11–0.14) |

[1] Color intensity represents value magnitude. Red indicates positive values, darker for larger. Blue indicates negative values, darker for more negative.

**Abbreviations:** ASPR, age-standardized Prevalence(per 100,000 population); UI, uncertainty interval; EAPC, estimated annual percentage change; CI, confidence interval; SDI, Socio-demographic Index.

# Table S2

Table S2 The YLDs burden of heart failure and its temporal trends by 204 countries and territories, for all ages and both sexes, 1990-2021

| | YLDs (95% UI) | | ASYR (95% UI) | | |
|---|---|---|---|---|---|
| | 1990 | 2021 | 1990 | 2021 | 1990–2021 EAPC (95% CI)[1] |
| **Central Asia** | | | | | |
| Armenia | 1750 (1152–2434) | 2819 (1820–3919) | 67.11 (43.15–93.96) | 71.62 (47.43–98.98) | 0.32 (0.26–0.38) |
| Azerbaijan | 3092 (2048–4417) | 5426 (3538–7677) | 60.79 (39.52–86.93) | 63.81 (41.77–91.03) | 0.23 (0.18–0.27) |
| Georgia | 3798 (2430–5390) | 3719 (2389–5320) | 66.91 (42.99–94.72) | 66.79 (42.98–95.17) | 0.02 (-0.02–0.07) |
| Kazakhstan | 7670 (5058–10847) | 10489 (6811–14791) | 62.08 (40.84–88.71) | 65.35 (42.18–92.74) | 0.28 (0.22–0.34) |
| Kyrgyzstan | 2080 (1377–2936) | 3077 (2020–4342) | 64.85 (42.93–91.72) | 65.31 (42.44–93.25) | 0.07 (0.03–0.11) |
| Mongolia | 753 (506–1064) | 1308 (861–1870) | 58.82 (38.27–83.99) | 61.22 (39.76–87.86) | 0.22 (0.18–0.27) |
| Tajikistan | 2034 (1352–2860) | 3726 (2484–5209) | 61.12 (39.34–86.08) | 62.61 (40.89–89.04) | 0.12 (0.08–0.17) |
| Turkmenistan | 1347 (913–1886) | 2380 (1553–3358) | 61.18 (40.06–86.41) | 63.69 (41.37–90.22) | 0.2 (0.16–0.24) |
| Uzbekistan | 8403 (5526–11654) | 14975 (9913–21423) | 60.41 (39.4–87.1) | 61.99 (40.12–88.87) | 0.12 (0.08–0.16) |
| **Central Europe** | | | | | |
| Albania | 1424 (943–1984) | 2490 (1576–3587) | 63.8 (41.52–89.65) | 64.32 (41.56–91.51) | 0.06 (0.04–0.08) |
| Bosnia and Herzegovina | 2285 (1505–3207) | 3374 (2160–4773) | 63.3 (41.45–89.2) | 63.69 (41.91–87.62) | 0.07 (0.05–0.1) |
| Bulgaria | 6199 (3972–9018) | 7399 (4591–10840) | 60.13 (39.3–85.95) | 58.76 (37.97–83.42) | -0.07 (-0.09–-0.05) |
| Croatia | 2824 (1823–4015) | 3743 (2433–5325) | 54.95 (36.11–77.5) | 49.12 (32.28–69.46) | -0.62 (-0.82–-0.43) |
| Czechia | 9488 (5915–13754) | 14533 (9258–20725) | 76.07 (48.55–109.44) | 76.3 (50.55–108.33) | 0.51 (0.25–0.76) |
| Hungary | 7901 (5094–11386) | 10714 (6752–15463) | 61.23 (40.39–87.47) | 61.96 (40.28–87.89) | 0.02 (0–0.03) |
| Montenegro | 387 (252–553) | 545 (343–781) | 66.25 (43.1–94.85) | 65.62 (42.45–92.5) | 0 (-0.02–0.01) |
| North Macedonia | 1058 (689–1476) | 1678 (1073–2392) | 61.37 (39.68–86.79) | 62.25 (40.21–87.21) | 0.07 (0.06–0.09) |
| Poland | 35897 (24215–49523) | 63408 (43324–87949) | 89.35 (60.11–123.73) | 96.71 (66.12–134.5) | 0.29 (0.18–0.39) |
| Romania | 14886 (9426–21109) | 20719 (13269–29970) | 60.82 (39.45–84.66) | 61.65 (40.82–87.41) | 0.32 (0.21–0.44) |
| Serbia | 4924 (3160–7004) | 7706 (4959–10992) | 52.31 (33.58–74.37) | 53.44 (35.34–74.63) | -0.43 (-0.64–-0.22) |
| Slovakia | 3519 (2242–5113) | 5229 (3319–7622) | 63.09 (40.55–90.63) | 63.72 (41.29–90.58) | 0.53 (0.37–0.7) |
| Slovenia | 1463 (932–2082) | 2685 (1761–3848) | 65.72 (42.69–93.13) | 67.95 (45.49–95.61) | 0.18 (0.15–0.21) |
| **Eastern Europe** | | | | | |
| Belarus | 7498 (4802–10582) | 9236 (5883–13359) | 64.2 (41.3–90.68) | 66.22 (42.94–94.18) | 0.17 (0.13–0.22) |
| Estonia | 1254 (809–1783) | 1704 (1082–2466) | 67.32 (44.22–94.98) | 68.74 (44.95–97.51) | 0.12 (0.09–0.15) |
| Latvia | 2156 (1352–3060) | 2438 (1558–3475) | 66.16 (42.68–93.34) | 66.58 (42.95–93.75) | 0.06 (0.02–0.11) |
| Lithuania | 2759 (1743–3929) | 3534 (2262–5038) | 65.87 (41.77–92.69) | 67.07 (44.27–94.19) | 0.09 (0.06–0.13) |
| Republic of Moldova | 2483 (1595–3562) | 3317 (2148–4754) | 62.43 (40.36–88.97) | 64.1 (41.81–90.72) | 0.13 (0.1–0.17) |
| Russian Federation | 101673 (68937–140926) | 137331 (92158–191646) | 64.33 (43.63–89.8) | 65.3 (44.39–90.42) | 0.09 (0.05–0.14) |
| Ukraine | 40653 (27062–56798) | 42572 (28472–60134) | 64.93 (43.75–89.95) | 63.14 (42.75–88.14) | -0.03 (-0.06–0) |
| **Australasia** | | | | | |
| Australia | 13565 (8834–19136) | 30868 (20747–43692) | 73.06 (48.12–102.75) | 73.26 (49.82–101.97) | -0.21 (-0.34–-0.07) |
| New Zealand | 2309 (1542–3239) | 4614 (3095–6384) | 61.64 (41.61–86.13) | 60.66 (41.22–83.46) | -0.19 (-0.28–-0.1) |
| **High-income Asia Pacific** | | | | | |
| Brunei Darussalam | 65 (44–90) | 136 (90–186) | 41.41 (27.78–57.33) | 42.06 (28.52–58.05) | 0.04 (0.03–0.04) |
| Japan | 64572 (43657–89456) | 135402 (92601–188307) | 45.56 (31.22–62.76) | 44.2 (30.13–60.91) | -0.14 (-0.17–-0.11) |
| Republic of Korea | 15361 (10422–21058) | 42961 (29227–58567) | 51.79 (35.26–72.02) | 56.59 (38.21–77.32) | 0.33 (0.21–0.46) |
| Singapore | 1038 (689–1422) | 3539 (2378–4927) | 47.48 (31.54–66.55) | 49.66 (33.36–68.87) | 0.14 (0.13–0.15) |
| **High-income North America** | | | | | |
| Canada | 25577 (16973–36333) | 55866 (35937–78749) | 83.14 (55.69–116.98) | 86.81 (57.2–120.29) | 0.16 (0.05–0.27) |
| Greenland | 23 (15–32) | 39 (25–55) | 69.51 (45.43–97.96) | 71.09 (46.46–99.28) | 0.11 (0.1–0.12) |
| United States of America | 241867 (163090–334578) | 429830 (296095–594497) | 79.69 (53.99–109.91) | 81.83 (55.92–113.19) | 0 (-0.03–0.04) |
| **Southern Latin America** | | | | | |
| Argentina | 18377 (12217–25711) | 28362 (18724–39621) | 58.34 (38.72–81.52) | 53.53 (35.64–74.27) | -0.27 (-0.29–-0.25) |
| Chile | 6241 (4185–8624) | 13401 (8869–18767) | 59.03 (39.53–81.86) | 57.66 (38.1–80) | -0.02 (-0.04–0) |
| Uruguay | 1925 (1264–2738) | 2709 (1785–3812) | 52.88 (34.7–74.08) | 52.29 (34.69–73.29) | -0.03 (-0.04–-0.02) |
| **Western Europe** | | | | | |
| Andorra | 26 (17–37) | 71 (45–102) | 52.33 (33.76–73.52) | 52.79 (34.51–74.52) | 0 (-0.02–0.01) |
| Austria | 9909 (6678–13891) | 11274 (7643–15706) | 87.31 (59.77–121.81) | 64.64 (44.42–89.14) | -1.33 (-1.47–-1.18) |
| Belgium | 6369 (4108–9344) | 8702 (5867–12120) | 46.09 (30.08–65.93) | 41.3 (28.24–57.15) | 0.02 (-0.09–0.13) |
| Cyprus | 305 (194–431) | 794 (498–1137) | 41.48 (27.1–58.96) | 43.91 (28.59–61.18) | -0.27 (-0.45–-0.1) |
| Denmark | 3165 (2035–4555) | 5098 (3257–7367) | 43.23 (28.98–61.3) | 47.45 (30.81–66.25) | 0.45 (0.22–0.68) |
| Finland | 3913 (2591–5529) | 7319 (4742–10496) | 60.76 (40.7–84.36) | 62.18 (41.31–87.73) | 0.12 (-0.09–0.33) |
| France | 79329 (52134–111723) | 142990 (94952–204933) | 100.11 (67.19–141.38) | 103.29 (67.32–143.42) | 0.04 (-0.09–0.16) |
| Germany | 68522 (44459–99326) | 113978 (72794–161491) | 60.36 (39.98–85.38) | 63.39 (40.85–88.58) | 0.97 (0.68–1.25) |
| Greece | 5824 (3730–8183) | 7132 (4732–10055) | 43.77 (28.86–61.06) | 34.03 (23.41–47.98) | -1.28 (-1.46–-1.1) |
| Iceland | 84 (55–118) | 156 (101–223) | 29.91 (19.66–41.51) | 30.07 (20.07–42.24) | -0.18 (-0.36–0.01) |
| Ireland | 1898 (1214–2721) | 3666 (2335–5197) | 48.61 (31.56–68.52) | 50.99 (33.07–71.31) | 0.18 (0.16–0.19) |
| Israel | 2072 (1357–2963) | 5662 (3643–7980) | 42.72 (28.43–60.89) | 45.98 (30.21–63.89) | 0.21 (0.13–0.29) |
| Italy | 51778 (34883–72091) | 94755 (65487–131070) | 66.25 (44.99–92.12) | 67.84 (46.6–93.66) | 0.25 (-0.01–0.5) |
| Luxembourg | 275 (176–391) | 564 (362–794) | 56.92 (37.47–80.16) | 58.69 (38.47–81.78) | -0.12 (-0.47–0.24) |
| Malta | 187 (121–269) | 439 (280–636) | 48.52 (32.3–68.67) | 49.79 (32.02–69.11) | 0.56 (0.38–0.74) |
| Monaco | 32 (21–47) | 46 (29–65) | 51.04 (33.8–72.58) | 51.63 (33.23–72.17) | 0.02 (0.02–0.03) |
| Netherlands | 8835 (5863–12618) | 16451 (10362–23701) | 47.53 (31.95–66.92) | 52.2 (33.68–73.77) | 0.21 (0.11–0.32) |
| Norway | 3389 (2296–4704) | 4968 (3327–7036) | 53.11 (35.95–73.84) | 54.4 (36.79–75.28) | 0.11 (0.09–0.12) |
| Portugal | 6139 (3866–8838) | 11679 (7377–16355) | 49.88 (32.21–70.84) | 50.87 (33.28–71.08) | -0.08 (-0.14–-0.02) |
| San Marino | 17 (11–25) | 38 (25–56) | 53.17 (34.63–75.44) | 53.58 (34.54–76.11) | 0 (-0.02–0.01) |
| Spain | 25507 (16097–36430) | 52502 (35385–72461) | 51.58 (32.98–72.33) | 57.26 (38.57–78.68) | 0.02 (-0.09–0.13) |
| Sweden | 12998 (8801–18202) | 20625 (13617–29278) | 97.27 (66.13–134.57) | 99.87 (66.88–137.67) | -0.03 (-0.11–0.05) |
| Switzerland | 4184 (2692–5921) | 7464 (4738–10523) | 43.53 (28.24–61.13) | 44.7 (28.94–61.74) | 0.39 (0.11–0.68) |
| United Kingdom | 33844 (22402–47730) | 46882 (31821–66296) | 41.06 (27.74–57.53) | 40.97 (27.87–57.04) | -0.17 (-0.22–-0.11) |

TableS2(continued）

| | | | | | |
|---|---|---|---|---|---|
| **Andean Latin America** | | | | | |
| Bolivia (Plurinational State of) | 2940 (1923–4220) | 6174 (4095–8624) | 78.39 (50.94–111.34) | 67.5 (44.82–92.91) | -0.32 (-0.38–-0.25) |
| Ecuador | 4053 (2739–5674) | 10728 (6970–15064) | 63.81 (41.86–90.53) | 65.55 (42.55–92.06) | 0.13 (0.12–0.15) |
| Peru | 8934 (6051–12378) | 23060 (15137–32041) | 63.58 (42.15–88.41) | 68.4 (44.64–95.68) | 0.29 (0.28–0.31) |
| **Caribbean** | | | | | |
| Antigua and Barbuda | 41 (27–58) | 64 (41–90) | 69.04 (45.99–97.96) | 69.53 (45.4–97.57) | -0.04 (-0.08–0) |
| Bahamas | 107 (71–150) | 232 (151–326) | 62.35 (40.67–88.45) | 65.74 (42.26–93.41) | 0.17 (0.16–0.18) |
| Barbados | 199 (129–287) | 321 (208–459) | 66.56 (43.89–94.43) | 70.82 (46.94–101.37) | 0.18 (0.17–0.19) |
| Belize | 79 (53–111) | 204 (135–282) | 64.34 (42.62–90.43) | 67.75 (44.11–95.54) | 0.16 (0.15–0.17) |
| Bermuda | 40 (26–57) | 91 (58–131) | 69.58 (45.98–97.97) | 76.16 (49.52–107.89) | 0.29 (0.27–0.31) |
| Cuba | 7007 (4513–9925) | 12588 (8051–18050) | 68.96 (44.41–96.96) | 72.25 (47.2–102.58) | 0.15 (0.13–0.16) |
| Dominica | 39 (26–56) | 47 (30–67) | 60.52 (39.66–86.63) | 63.7 (41.53–89.59) | 0.15 (0.14–0.16) |
| Dominican Republic | 2763 (1817–3864) | 6585 (4299–9187) | 63.64 (41.5–89.83) | 66.35 (43.11–92.64) | 0.1 (0.08–0.13) |
| Grenada | 52 (34–72) | 64 (41–91) | 60.95 (39.82–85.8) | 63.87 (42.55–90.34) | 0.09 (0.07–0.11) |
| Guyana | 261 (171–374) | 356 (235–501) | 56.93 (36.86–82.3) | 59.77 (39.22–84.69) | 0.13 (0.12–0.14) |
| Haiti | 1835 (1206–2553) | 4060 (2686–5594) | 52.33 (34.24–73.73) | 55.7 (35.15–78.43) | 0.19 (0.17–0.21) |
| Jamaica | 1382 (918–1963) | 2125 (1408–3004) | 67.57 (44.37–95.64) | 70.36 (46.34–99.06) | 0.07 (0.03–0.11) |
| Puerto Rico | 2532 (1615–3607) | 5096 (3272–7146) | 70.5 (45.15–99.12) | 75.57 (49.51–106.63) | 0.23 (0.22–0.24) |
| Saint Kitts and Nevis | 24 (16–35) | 36 (23–51) | 60.12 (39.06–85.35) | 64.89 (41.64–91.12) | 0.23 (0.2–0.26) |
| Saint Lucia | 65 (43–91) | 144 (94–202) | 64 (41.36–89.2) | 68.18 (45.21–95.62) | 0.19 (0.17–0.21) |
| Saint Vincent and the Grenadines | 51 (33–72) | 85 (54–119) | 61.64 (39.31–87.03) | 65.85 (42.41–92.85) | 0.2 (0.19–0.21) |
| Suriname | 170 (111–238) | 376 (245–533) | 62.47 (40.37–87.94) | 64.9 (42.56–91.81) | 0.11 (0.1–0.13) |
| Trinidad and Tobago | 571 (377–805) | 1150 (751–1641) | 62.5 (40.33–88.64) | 66.91 (43.74–94.43) | 0.24 (0.23–0.25) |
| United States Virgin Islands | 52 (33–71) | 109 (69–157) | 63.39 (40.53–89.24) | 67.75 (44.23–96.18) | 0.18 (0.17–0.19) |
| **Central Latin America** | | | | | |
| Colombia | 12493 (8319–17168) | 34848 (22649–49084) | 61.47 (40.65–85.82) | 65.61 (42.97–91.96) | 0.22 (0.21–0.24) |
| Costa Rica | 1420 (962–2001) | 3624 (2347–5110) | 67.09 (44.61–94.29) | 70.01 (45.31–98.05) | 0.17 (0.16–0.17) |
| El Salvador | 2236 (1505–3132) | 4299 (2862–6063) | 62.33 (41.18–87.69) | 66.09 (43.73–92.62) | 0.25 (0.23–0.26) |
| Guatemala | 2690 (1790–3745) | 7241 (4800–9991) | 59.21 (39.25–83.73) | 61.78 (40.39–86.56) | 0.18 (0.16–0.19) |
| Honduras | 1619 (1081–2268) | 4093 (2724–5686) | 59.74 (39.39–83.96) | 60.34 (38.81–84.71) | 0.07 (0.06–0.09) |
| Mexico | 34258 (23123–47222) | 80781 (54609–112072) | 65.8 (44.41–90.4) | 67.27 (45.71–93.34) | 0.08 (0.06–0.09) |
| Nicaragua | 1400 (940–1955) | 3411 (2280–4683) | 64.67 (42.32–90.17) | 67.09 (43.96–92.21) | 0.16 (0.14–0.17) |
| Panama | 1121 (749–1579) | 3054 (2018–4231) | 66.77 (43.64–93.74) | 69.33 (45.88–96.15) | 0.13 (0.11–0.14) |
| Venezuela (Bolivarian Republic of) | 7673 (5200–10621) | 18590 (12163–25795) | 65.88 (43.53–92.43) | 67.03 (43.98–92.84) | 0.13 (0.11–0.14) |
| **Tropical Latin America** | | | | | |
| Brazil | 61956 (41667–84916) | 149769 (101823–206747) | 63.53 (42.85–87.35) | 64.27 (43.7–88.49) | 0.08 (0.06–0.1) |
| Paraguay | 1749 (1195–2463) | 3834 (2503–5376) | 64.33 (42.78–91.06) | 65.11 (42.05–91.13) | 0.07 (0.06–0.09) |
| **North Africa and Middle East** | | | | | |
| Afghanistan | 4085 (2693–5659) | 8982 (6059–12609) | 58.02 (38.22–82.01) | 58.67 (38.33–81.89) | 0.04 (0.03–0.06) |
| Algeria | 10807 (7217–15285) | 26494 (17287–36700) | 71.41 (46.88–99.43) | 74.46 (48.94–104.45) | 0.15 (0.14–0.16) |
| Bahrain | 182 (121–254) | 755 (515–1050) | 70.06 (46.08–98.37) | 76.67 (51.13–106.72) | 0.32 (0.31–0.33) |
| Egypt | 21225 (14318–29489) | 46901 (31527–64712) | 66.29 (43.93–93.46) | 70.27 (46.58–97.06) | 0.18 (0.17–0.19) |
| Iran (Islamic Republic of) | 23919 (16351–33261) | 56127 (38639–76381) | 72.21 (49.55–99.83) | 75.52 (52.12–103.73) | 0.13 (0.12–0.14) |
| Iraq | 7583 (5050–10638) | 18820 (12468–26162) | 68.28 (44.53–96.4) | 71.18 (47.42–99.06) | 0.15 (0.14–0.16) |
| Jordan | 1451 (990–2011) | 6235 (4153–8628) | 73.27 (48.53–102.95) | 77.04 (51.06–106.42) | 0.18 (0.17–0.2) |
| Kuwait | 692 (461–963) | 2594 (1717–3624) | 79.9 (52.3–112.22) | 83.49 (54.67–114.95) | 0.16 (0.12–0.2) |
| Lebanon | 1636 (1079–2272) | 4643 (3039–6488) | 72.94 (47.78–102.72) | 77.52 (50.67–108.24) | 0.21 (0.19–0.23) |
| Libya | 1819 (1233–2546) | 3736 (2491–5228) | 72.71 (47.63–102.05) | 73.18 (48.42–102.61) | 0.06 (0.04–0.07) |
| Morocco | 10936 (7286–15200) | 22184 (14780–30928) | 65.3 (42.9–91.26) | 68.2 (44.87–94.87) | 0.14 (0.14–0.14) |
| Oman | 695 (476–970) | 1877 (1262–2593) | 64.01 (43.09–88.67) | 70.77 (47.45–99.42) | 0.32 (0.3–0.34) |
| Palestine | 866 (582–1216) | 2274 (1511–3178) | 68.73 (44.92–95.76) | 71.91 (46.87–99.46) | 0.14 (0.13–0.15) |
| Qatar | 157 (105–218) | 1215 (817–1752) | 75 (49.15–104.12) | 81.69 (53.93–113.65) | 0.33 (0.31–0.36) |
| Saudi Arabia | 5940 (4003–8304) | 15778 (10324–21884) | 69.35 (45.61–96.85) | 75.63 (49.48–105.87) | 0.3 (0.29–0.31) |
| Sudan | 7209 (4733–10144) | 16080 (10763–22378) | 62.67 (40.76–89.17) | 65.69 (43.61–91.43) | 0.16 (0.15–0.16) |
| Syrian Arab Republic | 5124 (3497–7119) | 8830 (5770–12367) | 70.01 (45.96–97.97) | 73.88 (48.77–103.31) | 0.18 (0.15–0.21) |
| Tunisia | 4048 (2639–5722) | 9153 (5950–12692) | 72.42 (46.84–101.56) | 75.17 (48.95–103.97) | 0.11 (0.11–0.12) |
| Türkiye | 28524 (19025–39825) | 67825 (44361–93133) | 74.41 (49.54–103.65) | 78.52 (51.49–107.12) | 0.21 (0.19–0.23) |
| United Arab Emirates | 634 (421–885) | 4287 (2844–6069) | 74.11 (48–103.23) | 80.44 (53.54–112.46) | 0.27 (0.25–0.29) |
| Yemen | 4351 (2937–6115) | 11752 (7896–16362) | 61.16 (40.16–85.72) | 63.55 (41.08–88.99) | 0.14 (0.12–0.16) |
| **South Asia** | | | | | |
| Bangladesh | 32618 (22500–45337) | 78325 (51079–107523) | 52.04 (34.45–72.68) | 58.2 (38.28–79.97) | 0.38 (0.36–0.39) |
| Bhutan | 164 (113–231) | 346 (227–484) | 50.2 (32.93–70.78) | 56.74 (37.13–78.96) | 0.43 (0.42–0.44) |
| India | 253150 (172880–347081) | 624997 (425464–854741) | 48.98 (33.5–66.79) | 54.96 (37.44–75.83) | 0.43 (0.41–0.45) |
| Nepal | 5390 (3591–7544) | 12587 (8316–17420) | 48.26 (31.56–67.86) | 54.22 (35.98–74.81) | 0.39 (0.38–0.4) |
| Pakistan | 35086 (23542–48300) | 75369 (51162–103772) | 50.42 (34.16–69.94) | 53.49 (36.24–74.1) | 0.21 (0.19–0.22) |
| **East Asia** | | | | | |
| China | 459520 (313552–630785) | 1290810 (865894–1775731) | 62.83 (42.81–87.18) | 67.79 (45.92–93.34) | 0.24 (0.2–0.28) |
| Democratic People's Republic of Korea | 7358 (4781–10363) | 16854 (10924–23869) | 52.96 (34.41–75.6) | 58.18 (37.92–82.88) | 0.3 (0.29–0.32) |
| Taiwan (Province of China) | 8942 (5809–12903) | 25555 (16386–36588) | 62.15 (40.41–88.83) | 65.23 (42.19–92.48) | 0.12 (0.03–0.22) |
| **Oceania** | | | | | |
| American Samoa | 14 (9–19) | 23 (15–33) | 53.84 (34.52–76.82) | 55.99 (36.66–78.83) | 0.1 (0.09–0.11) |
| Cook Islands | 7 (5–10) | 14 (9–20) | 54.69 (36.07–77.59) | 58.49 (37.89–85.02) | 0.21 (0.21–0.21) |
| Fiji | 192 (122–269) | 346 (227–485) | 49.72 (31.96–70.51) | 51.59 (34.18–72.37) | 0.08 (0.07–0.1) |
| Guam | 42 (28–59) | 116 (76–165) | 56.07 (36.48–80.07) | 57.99 (37.52–82.28) | 0.1 (0.08–0.13) |
| Kiribati | 18 (11–25) | 32 (21–44) | 45.75 (29.64–64.84) | 47.64 (31.23–66.72) | 0.11 (0.1–0.12) |
| Marshall Islands | 11 (7–15) | 16 (10–22) | 48.1 (31.84–68.02) | 50.7 (33.43–71.45) | 0.18 (0.17–0.19) |
| Micronesia (Federated States of) | 28 (18–39) | 32 (21–45) | 48.11 (30.89–68.48) | 50.7 (32.22–71.59) | 0.16 (0.14–0.17) |

TableS2(continued）

| | | | | | |
|---|---|---|---|---|---|
| Nauru | 2 (2–3) | 3 (2–4) | 48.52 (31.53–69.67) | 49.34 (32.03–69.23) | 0.05 (0.01–0.09) |
| Niue | 1 (1–2) | 1 (1–1) | 51.61 (33.5–72.91) | 54.53 (35.8–76.11) | 0.15 (0.15–0.16) |
| Northern Mariana Islands | 11 (8–16) | 25 (16–35) | 60 (39.07–85.1) | 60.69 (39.98–86.18) | -0.03 (-0.05–-0.01) |
| Palau | 5 (3–7) | 9 (6–14) | 52.12 (33.53–75.33) | 55.02 (36.11–79.58) | 0.15 (0.14–0.16) |
| Papua New Guinea | 923 (607–1277) | 2565 (1705–3581) | 49.44 (32.16–69.44) | 50.96 (32.9–71.66) | 0.06 (0.05–0.08) |
| Samoa | 51 (34–72) | 81 (54–113) | 52.82 (33.92–75.5) | 55.04 (36.6–78.23) | 0.1 (0.09–0.11) |
| Solomon Islands | 77 (51–109) | 179 (119–254) | 48.47 (31.14–68.69) | 49.98 (32.93–69.83) | 0.07 (0.06–0.09) |
| Tokelau | 1 (0–1) | 1 (1–1) | 51.9 (33.15–72.74) | 54.86 (35.4–78.42) | 0.16 (0.15–0.17) |
| Tonga | 33 (22–47) | 48 (31–66) | 54.9 (35.8–77.94) | 56.47 (35.87–78.23) | 0.07 (0.06–0.08) |
| Tuvalu | 3 (2–4) | 5 (3–7) | 47.66 (31.07–68.31) | 50.84 (33.35–72.49) | 0.18 (0.17–0.2) |
| Vanuatu | 36 (24–52) | 87 (57–122) | 49.08 (32.16–70.51) | 49.88 (32.39–70.62) | 0.04 (0.03–0.04) |
| **Southeast Asia** | | | | | |
| Cambodia | 2408 (1610–3402) | 5769 (3752–8123) | 46.69 (30.29–66.12) | 50.75 (33.28–73.13) | 0.28 (0.27–0.3) |
| Indonesia | 50573 (34714–69097) | 109258 (73519–150497) | 50.66 (34.73–71.12) | 53.75 (36.33–74.86) | 0.17 (0.16–0.19) |
| Lao People's Democratic Republic | 999 (652–1402) | 2180 (1428–3086) | 45.48 (29.22–64.37) | 49.49 (32.29–70.8) | 0.28 (0.26–0.3) |
| Malaysia | 5791 (3850–8053) | 15209 (9873–21403) | 53.73 (34.86–76.41) | 58.43 (38.27–81.69) | 0.28 (0.27–0.29) |
| Maldives | 58 (39–81) | 193 (130–269) | 54.45 (35.37–78.06) | 59.6 (39.12–83.63) | 0.3 (0.29–0.31) |
| Mauritius | 395 (260–555) | 916 (602–1306) | 54.56 (35.14–76.14) | 57.89 (38.22–81.49) | 0.15 (0.14–0.17) |
| Myanmar | 10602 (6944–14862) | 22169 (14210–31775) | 46.78 (30.53–65.93) | 50.4 (32.59–72.5) | 0.27 (0.25–0.29) |
| Philippines | 17243 (11773–23739) | 40972 (28166–56498) | 51.23 (34.83–71.41) | 52.5 (35.78–73.6) | 0.06 (0.05–0.07) |
| Seychelles | 32 (21–45) | 56 (36–80) | 51.61 (33.34–73.11) | 55.65 (35.86–78.46) | 0.25 (0.24–0.26) |
| Sri Lanka | 5876 (3864–8223) | 14326 (9256–20507) | 54.4 (35.28–76.19) | 59 (38.34–84.77) | 0.29 (0.27–0.3) |
| Thailand | 20029 (13117–28446) | 61732 (39162–87420) | 58.1 (37.5–83.28) | 63.29 (40.54–89.51) | 0.28 (0.27–0.28) |
| Timor-Leste | 160 (108–224) | 460 (303–639) | 48.18 (30.99–67.14) | 51.05 (33.31–71.79) | 0.19 (0.18–0.2) |
| Viet Nam | 23668 (15934–32904) | 51877 (33818–72986) | 53.61 (35.68–75.73) | 59.17 (37.96–82.5) | 0.32 (0.31–0.32) |
| **Central Sub-Saharan Africa** | | | | | |
| Angola | 3122 (2056–4371) | 10586 (7118–14845) | 65.76 (43.07–93.73) | 70.06 (45.67–98.39) | 0.22 (0.2–0.23) |
| Central African Republic | 781 (528–1085) | 1581 (1057–2228) | 60.68 (39.13–85.59) | 63.19 (41.23–89.56) | 0.13 (0.13–0.14) |
| Congo | 824 (541–1153) | 2044 (1365–2853) | 65.48 (42.97–94.43) | 70.66 (46.14–100.33) | 0.26 (0.24–0.27) |
| Democratic Republic of the Congo | 12307 (8081–17197) | 30523 (20788–43185) | 66.39 (42.68–95.53) | 69.49 (44.06–98.88) | 0.14 (0.12–0.17) |
| Equatorial Guinea | 145 (95–204) | 494 (333–698) | 64.91 (42.5–90.83) | 72.22 (46.31–100.76) | 0.39 (0.37–0.4) |
| Gabon | 439 (288–612) | 802 (531–1128) | 68.32 (44.36–96.51) | 72.57 (47.29–101.37) | 0.2 (0.19–0.21) |
| **Eastern Sub-Saharan Africa** | | | | | |
| Burundi | 2005 (1317–2848) | 4513 (2975–6331) | 70.58 (47.23–101.32) | 74.4 (48.6–104.92) | 0.17 (0.16–0.18) |
| Comoros | 181 (119–253) | 394 (262–550) | 75.87 (49.4–108.61) | 78.59 (51.77–109.65) | 0.1 (0.09–0.12) |
| Djibouti | 134 (90–187) | 533 (356–731) | 75.51 (48.2–106.89) | 78.86 (51.13–109.85) | 0.14 (0.12–0.16) |
| Eritrea | 987 (656–1376) | 2320 (1546–3210) | 69 (44.92–99.54) | 72.43 (46.3–101.43) | 0.12 (0.11–0.14) |
| Ethiopia | 16798 (11246–23376) | 42424 (28657–58906) | 69.13 (46.58–95.61) | 77.13 (51.8–106.96) | 0.36 (0.35–0.37) |
| Kenya | 8720 (5882–12097) | 20829 (14064–28439) | 75.76 (51.65–104.26) | 77.4 (52.93–107.27) | -0.38 (-0.55–-0.22) |
| Madagascar | 4519 (2996–6240) | 10246 (6835–14214) | 74.55 (47.76–105.03) | 77.29 (49.57–111.78) | 0.1 (0.09–0.1) |
| Malawi | 3278 (2130–4585) | 6779 (4604–9614) | 70.13 (45.6–98.91) | 73.9 (48.4–104.69) | 0.14 (0.12–0.15) |
| Mozambique | 4991 (3268–6882) | 10780 (7211–15093) | 73.54 (47.91–104.68) | 76.72 (49.58–107.8) | 0.09 (0.07–0.1) |
| Rwanda | 2442 (1622–3420) | 5292 (3510–7320) | 70.85 (46.15–101.05) | 75.17 (48.92–105.18) | 0.19 (0.17–0.21) |
| Somalia | 2161 (1423–3012) | 5960 (3957–8352) | 67.66 (43.68–96.11) | 68.44 (45.57–96.13) | 0.02 (0–0.03) |
| South Sudan | 2232 (1461–3160) | 3458 (2325–4806) | 72.53 (47.46–102.95) | 75.08 (48.25–104.72) | 0.09 (0.09–0.1) |
| Uganda | 5998 (3965–8418) | 14800 (9929–20712) | 71.75 (45.87–100.76) | 74.35 (48.25–104.31) | 0.08 (0.06–0.1) |
| United Republic of Tanzania | 9719 (6247–13498) | 23751 (16069–33439) | 73.22 (47.03–104.07) | 77.02 (50.74–107.85) | 0.37 (0.29–0.45) |
| Zambia | 2564 (1681–3559) | 6584 (4437–9320) | 70.25 (45.44–99.5) | 74.28 (47.9–104.77) | 0.16 (0.14–0.19) |
| **Southern Sub-Saharan Africa** | | | | | |
| Botswana | 474 (311–670) | 1052 (710–1468) | 69.42 (45.15–97.97) | 71.4 (46.2–100.7) | 0.08 (0.05–0.11) |
| Eswatini | 258 (172–364) | 421 (282–591) | 66.23 (42.78–94.59) | 68.35 (44.38–96.31) | 0.09 (0.06–0.12) |
| Lesotho | 623 (416–871) | 738 (489–1038) | 64.91 (42.17–93.72) | 66.19 (42.96–92.8) | 0.04 (0.01–0.07) |
| Namibia | 541 (359–770) | 1083 (716–1540) | 71.05 (46.26–101.91) | 74.15 (48.65–104.65) | 0.13 (0.11–0.15) |
| South Africa | 16957 (11405–23430) | 32973 (22175–45725) | 72.53 (49.32–101.55) | 74.51 (50.7–104) | 0.1 (0.07–0.13) |
| Zimbabwe | 3553 (2341–5014) | 5481 (3711–7762) | 67.51 (43.54–97.02) | 68.09 (44.59–96.88) | -0.03 (-0.05–0) |
| **Western Sub-Saharan Africa** | | | | | |
| Benin | 1799 (1170–2544) | 4827 (3299–6612) | 68.25 (44.55–96.88) | 71.57 (46.93–100.05) | 0.14 (0.13–0.15) |
| Burkina Faso | 3338 (2214–4741) | 7934 (5329–11145) | 65.31 (42.85–93.47) | 67.99 (44.23–95.99) | 0.14 (0.12–0.15) |
| Cabo Verde | 219 (145–308) | 365 (242–512) | 78.1 (51.43–111.42) | 82.84 (54.27–117.43) | 0.2 (0.18–0.22) |
| Cameroon | 3645 (2450–5122) | 11276 (7547–15760) | 67.47 (43.67–96.82) | 72.42 (47.33–102.2) | 0.21 (0.19–0.22) |
| Chad | 2256 (1492–3185) | 5565 (3747–7787) | 65.54 (43.1–93.59) | 68.89 (45.23–96.67) | 0.17 (0.15–0.18) |
| Côte d'Ivoire | 3637 (2446–5060) | 9782 (6641–13706) | 67.77 (43.77–97.12) | 71.82 (46.82–99.94) | 0.19 (0.17–0.21) |
| Gambia | 334 (221–468) | 914 (609–1266) | 69.53 (45.89–99.5) | 72.96 (47.71–102.64) | 0.16 (0.15–0.17) |
| Ghana | 5110 (3398–7044) | 13246 (8773–18468) | 66.32 (42.91–93.47) | 69.84 (44.56–98.15) | 0.15 (0.14–0.16) |
| Guinea | 2479 (1629–3463) | 4883 (3187–6757) | 66.39 (43–94.38) | 69.8 (45.17–97.12) | 0.15 (0.14–0.16) |
| Guinea-Bissau | 310 (205–428) | 618 (418–875) | 63.47 (41.17–89.92) | 67.28 (44.55–96.65) | 0.2 (0.18–0.21) |
| Liberia | 937 (614–1315) | 1890 (1245–2623) | 67.56 (43.78–96.39) | 72.38 (47.23–101.79) | 0.22 (0.22–0.23) |
| Mali | 2989 (1985–4167) | 7970 (5366–11354) | 66 (41.99–93.82) | 70.04 (46.14–99.45) | 0.18 (0.17–0.19) |
| Mauritania | 863 (579–1233) | 1966 (1311–2751) | 70.81 (46.78–99.58) | 76.3 (49.65–107.17) | 0.25 (0.23–0.26) |
| Niger | 2395 (1627–3347) | 7813 (5310–11067) | 66.35 (43.38–94.84) | 69.82 (45.84–98.36) | 0.18 (0.16–0.19) |
| Nigeria | 37265 (25062–51438) | 87006 (58958–120221) | 71.76 (48.87–99.94) | 74.68 (50.5–103.7) | 0.1 (0.08–0.12) |
| Sao Tome and Principe | 55 (37–78) | 95 (64–135) | 70.87 (45.84–101.35) | 76.16 (49.32–106.7) | 0.23 (0.22–0.24) |
| Senegal | 2897 (1919–4109) | 6698 (4450–9333) | 70.41 (46.34–99.02) | 74.26 (48.14–104.01) | 0.15 (0.14–0.16) |
| Sierra Leone | 1628 (1084–2330) | 3243 (2171–4526) | 67.27 (44.13–96.95) | 71.3 (47.28–99.77) | 0.17 (0.16–0.18) |
| Togo | 1156 (780–1609) | 3076 (2068–4240) | 68.03 (43.93–96.22) | 71.36 (46.58–99.2) | 0.14 (0.13–0.15) |

† Color intensity represents value magnitude. Red indicates positive values, darker for larger. Blue indicates negative values, darker for more negative.

**Abbreviations:** YLDs, years lived with disability; ASYR, Age-standardised YLDs rate(per 100,000 population); UI, uncertainty interval; EAPC, estimated annual percentage change; CI, confidence interval; SDI, Socio-demographic Index.
